# DNA methylation age calculators reveal association with diabetic neuropathy in type 1 diabetes

**DOI:** 10.1186/s13148-020-00840-6

**Published:** 2020-04-05

**Authors:** Delnaz Roshandel, Zhuo Chen, Angelo J. Canty, Shelley B. Bull, Rama Natarajan, Andrew D. Paterson, DCCT/EDIC Research Group, S. Scherer, F. Miao, L. Zhang, J. Brown-Friday, J. Crandall, H. Engel, S. Engel, H. Martinez, M. Phillips, M. Reid, H. Shamoon, J. Sheindlin, R. Gubitosi-Klug, J. Wood, L. Mayer, D. Miller, A. Nayate, M. Novak, S. Pendegast, L. Singerman, D. Weiss, H. Zegarra, E. Brown, P. Crawford, S. Genuth, M. Palmert, P. Pugsley, J. Quin, S. Smith-Brewer, W. Dahms, J. McConnell, C. Beck, K. Farrell, P. Gaston, R. Trail, V. Monnier, D. Sell, C. Strauch, N. S. Gregory, R. Hanna, R. Chan, S. Kiss, A. Orlin, M. Rubin, S. Barron, B. Bosco, D. Brillon, S. Chang, A. Dwoskin, M. Heinemann, L. Jovanovic, M. E. Lackaye, T. Lee, B. Levy, V. Reppucci, M. Richardson, R. Campbell, A. Bhan, J. K. Jones, D. Kruger, P. A. Edwards, H. Remtema, E. Angus, A. Galprin, M. McLellan, A. Thomas, J. D. Carey, F. Whitehouse, R. Bergenstal, S. Dunnigan, K. Gunyou, M. Johnson, A. Carlson, L. Thomas, R. Birk, P. Callahan, G. Castle, R. Cuddihy, M. Franz, D. Freking, L. Gill, J. Gott, P. Hollander, D. Kendall, J. Laechelt, S. List, W. Mestrezat, J. Nelson, B. Olson, N. Rude, M. Spencer, D. Etzwiler, K. Morgan, L. P. Aiello, E. Golden, P. Arrigg, R. Beaser, L. Bestourous, J. Cavallerano, R. Cavicchi, O. Ganda, O. Hamdy, T. Murtha, D. Schlossman, S. Shah, G. Sharuk, P. Silva, P. Silver, M. Stockman, J. Sun, E. Weimann, L. M. Aiello, V. Asuquo, A. Jacobson, R. Kirby, L. Rand, J. Rosenzwieg, H. Wolpert, D. M. Nathan, M. E. Larkin, M. Cayford, A. de Manbey, L. Gurry, J. Heier, A. Joseph, F. Leandre, K. Martin, C. Shah, C. Stevens, N. Thangthaeng, E. Anderson, H. Bode, S. Brink, M. Christofi, C. Cornish, D. Cros, S. Crowell, L. Delahanty, K. Folino, S. Fritz, C. Gauthier-Kelly, J. Godine, C. Haggan, K. Hansen, P. Lou, J. Lynch, C. McKitrick, D. Moore, D. Norman, M. Ong, E. Ryan, C. Taylor, D. Zimbler, A. Vella, G. Ziegler, A. Barkmeier, B. French, M. Haymond, J. Mortenson, J. Pach, R. Rizza, L. Schmidt, W. F. Schwenk, F. J. Service, R. Woodwick, R. Colligan, A. Lucas, B. Zimmerman, K. Nickander, P. Low, C. Sommer, M. Lopes-Virella, L. Luttrell, L. Spillers, J. Fernandes, K. Hermayer, S. Kwon, K. Lee, T. Lyons, M. Nutaitis, A. Blevins, M. Bracey, S. Caulder, J. Colwell, S. Elsing, A. Farr, D. Lee, P. Lindsey, R. Mayfield, J. Parker, N. Patel, C. Pittman, J. Selby, J. Soule, M. Szpiech, T. Thompson, D. Wood, S. Yacoub-Wasef, A. Jenkins, R. Klein, G. Virella, A. Jaffa, R. Carter, J. Stoner, W. T. Garvey, D. Lackland, M. Brabham, D. McGee, D. Zheng, R. K. Mayfield, M. Molitch, A. Wallia, D. Adelman, M. Hartmuller, S. Ajroud-Driss, P. Astelford, A. Degillio, M. Gill, L. Jampol, C. Johnson, L. Kaminski, N. Leloudes, A. Lyon, R. Mirza, D. Ryan, E. Simjanoski, Z. Strugula, S. Colson, B. Schaefer, O. Kolterman, S. Mudaliar, G. Lorenzi, M. Goldbaum, T. Clark, M. Giotta, I. Grant, K. Jones, R. Lyon, M. Prince, R. Reed, M. Swenson, G. Friedenberg, W. I. Sivitz, B. Vittetoe, J. Kramer, M. Bayless, C. Fountain, R. Hoffman, J. MacIndoe, N. Olson, H. Schrott, L. Snetselaar, T. Weingeist, R. Zeitler, R. Miller, S. Johnsonbaugh, M. Carney, D. Counts, T. Donner, J. Gordon, M. Hebdon, R. Hemady, B. Jones, A. Kowarski, R. Liss, S. Mendley, D. Ostrowski, M. Patronas, P. Salemi, S. Steidl, W. H. Herman, C. L. Martin, P. Lee, J. W. Albers, R. Pop-Busui, E. L. Feldman, D. A. Greene, M. J. Stevens, N. Burkhart, T. Sandford, J. Floyd, A. Sarma, R. Dunn, T. Speigelberg, J. Bantle, N. Flaherty, D. Koozekanani, S. Montezuma, J. Terry, F. Goetz, C. Kwong, L. McKenzie, M. Mech, J. Olson, B. Rogness, T. Strand, R. Warhol, N. Wimmergren, M. Steffes, A. Karger, J. Seegmiller, V. Arends, J. Bucksa, B. Chavers, A. Killeen, M. Nowicki, A. Saenger, R. Crow, B. Gloeb, C. O’Donnell, S. Thomas, D. Goldstein, D. Hainsworth, S. Hitt, J. Giangiacomo, D. S. Schade, J. L. Canady, R. B. Avery, M. R. Burge, J. E. Chapin, A. Das, L. H. Ketai, D. Hornbeck, C. Johannes, J. Rich, M. L. Schluter, M. Schutta, P. A. Bourne, A. Brucker, S. Braunstein, B. J. Maschak-Carey, S. Schwartz, L. Baker, T. Orchard, L. Cimino, D. Rubinstein, D. Becker, B. Doft, D. Finegold, K. Kelly, L. Lobes, N. Silvers, T. Songer, D. Steinberg, L. Steranchak, J. Wesche, A. Drash, C. Ryan, B. Burzuk, E. Cupelli, M. Geckle, D. Sandstrom, F. Thoma, T. Williams, T. Woodfill, J. I. Malone, A. Morrison, M. L. Bernal, P. R. Pavan, L. Babbione, T. J. DeClue, N. Grove, D. McMillan, H. Solc, E. A. Tanaka, J. Vaccaro-Kish, S. Dagogo-Jack, C. Wigley, S. Huddleston, A. Patel, M. Bryer-Ash, E. Chaum, A. Iannacone, H. Lambeth, D. Meyer, S. Moser, M. B. Murphy, H. Ricks, S. Schussler, S. Yoser, A. Kitabchi, P. Raskin, S. Strowig, Y. G. He, E. Mendelson, R. L. Ufret-Vincenty, M. Basco, S. Cercone, L. Sun, A. Boright, B. A. Perkins, B. Zinman, A. Barnie, N. Bakshi, M. Brent, R. Devenyi, K. Koushan, M. Mandelcorn, F. Perdikaris, L. Tuason, D. Daneman, R. Ehrlich, S. Ferguson, A. Gordon, K. Perlman, S. Rogers, J. Palmer, R. Fahlstrom, I. H. de Boer, L. Olmos de Koo, L. Van Ottingham, S. Catton, J. Ginsberg, J. Kinyoun, J. Brunzell, J. Purnell, H. Wessells, S. Holt, J. Hotaling, C. Kim, Q. Clemens, J. Brown, C. McDonald, M. Driscoll, J. Bylsma, T. Sheidow, W. Brown, C. Canny, P. Colby, S. Debrabandere, J. Dupre, J. Harth, I. Hramiak, M. Jenner, J. Mahon, D. Nicolle, N. W. Rodger, T. Smith, O. Crofford, M. May, J. Lipps Hagan, R. Ramker, T. Adkins, A. Agarwal, C. Lovell, S. Feman, R. Lorenz, L. Survant, N. H. White, L. Levandoski, I. Boniuk, J. Santiago, W. Tamborlane, P. Gatcomb, K. Stoessel, J. Ahern, K. Fong, P. Ossorio, P. Ramos, J. Lachin, I. Bebu, B. Braffett, J. Backlund, L. Diminick, X. Gao, D. Kenny, K. Klumpp, M. Lin, V. Trapani, K. Anderson, K. Chan, P. Cleary, A. Determan, L. Dews, W. Hsu, P. McGee, H. Pan, B. Petty, D. Rosenberg, B. Rutledge, W. Sun, S. Villavicencio, N. Younes, C. Williams, C. Cowie, C. Siebert, A. Harrington, D. O’Leary, J. Polak, L. Funk, E. Z. Soliman, M. Barr, C. Campbell, S. Hensley, J. Hu, L. Keasler, Y. Li, T. Taylor, Z. M. Zhang, R. Prineas, B. Blodi, R. Danis, D. Lawrence, H. Wabers, M. Burger, M. Davis, J. Dingledine, V. Gama, S. Gangaputra, L. Hubbard, S. Neill, R. Sussman, K. Cruickshanks, D. Dalton, K. Bainbridge, M. Budoff, S. Darabian, P. Rezaeian, R. Detrano, M. Fox, L. Kim, R. Oudiz, N. Wong, J. Lima, D. Bluemke, E. Turkbey, H. Chahal, A. Jain, R. Jarboe, C. Liu, A. Malayeri, C. Miao, R. J. van der Geest, S. Hazen, A. Pratt, W. Tang, J. Maynard, J. Farquhar, M. Moran, M. Pfiefer, M. Schumer, K. McVary

**Affiliations:** 1 Genetics and Genome Biology Program, The Hospital for Sick Children, Toronto, ON Canada; 2 Department of Diabetes Complications and Metabolism, Beckman Research Institute of City of Hope, Duarte, CA USA; 3 Department of Mathematics and Statistics, McMaster University, Hamilton, ON Canada; 4 Lunenfeld–Tanenbaum Research Institute, Sinai Health System, Toronto, ON Canada; 5 Dalla Lana School of Public Health, University of Toronto, Toronto, ON Canada

**Keywords:** DNA methylation age, Type 1 diabetes, Diabetic complications

## Abstract

**Background:**

Many CpGs become hyper or hypo-methylated with age. Multiple methods have been developed by Horvath et al. to estimate DNA methylation (DNAm) age including Pan-tissue, Skin & Blood, PhenoAge, and GrimAge. Pan-tissue and Skin & Blood try to estimate chronological age in the normal population whereas PhenoAge and GrimAge use surrogate markers associated with mortality to estimate biological age and its departure from chronological age. Here, we applied Horvath’s four methods to calculate and compare DNAm age in 499 subjects with type 1 diabetes (T1D) from the Diabetes Control and Complications Trial/Epidemiology of Diabetes Interventions and Complications (DCCT/EDIC) study using DNAm data measured by Illumina EPIC array in the whole blood. Association of the four DNAm ages with development of diabetic complications including cardiovascular diseases (CVD), nephropathy, retinopathy, and neuropathy, and their risk factors were investigated.

**Results:**

Pan-tissue and GrimAge were higher whereas Skin & Blood and PhenoAge were lower than chronological age (*p* < 0.0001). DNAm age was not associated with the risk of CVD or retinopathy over 18–20 years after DNAm measurement. However, higher PhenoAge (*β* = 0.023, *p* = 0.007) and GrimAge (*β* = 0.029, *p* = 0.002) were associated with higher albumin excretion rate (AER), an indicator of diabetic renal disease, measured over time. GrimAge was also associated with development of both diabetic peripheral neuropathy (OR = 1.07, *p* = 9.24E−3) and cardiovascular autonomic neuropathy (OR = 1.06, *p* = 0.011). Both HbA1c (*β* = 0.38, *p* = 0.026) and T1D duration (*β* = 0.01, *p* = 0.043) were associated with higher PhenoAge. Employment (*β* = − 1.99, *p* = 0.045) and leisure time (*β* = − 0.81, *p* = 0.022) physical activity were associated with lower Pan-tissue and Skin & Blood, respectively. BMI (*β* = 0.09, *p* = 0.048) and current smoking (*β* = 7.13, *p* = 9.03E−50) were positively associated with Skin & Blood and GrimAge, respectively. Blood pressure, lipid levels, pulse rate, and alcohol consumption were not associated with DNAm age regardless of the method used.

**Conclusions:**

Various methods of measuring DNAm age are sub-optimal in detecting people at higher risk of developing diabetic complications although some work better than the others.

## Background

CpGs are regions of DNA where a cytosine is followed by a guanine nucleotide. Cytosines within CpGs can be methylated, and CpG methylation levels affect gene expression. Many CpGs become hyper or hypo-methylated with age [1–4]. In 2013, Horvath used publicly available DNA methylation (DNAm) data to define and evaluate a DNAm age predictor, Pan-tissue, which is accurate across most tissues and cell types. Chronological age was regressed on CpG methylation levels using a penalized regression model (elastic net) which selected 353 CpGs [1] (Supplementary Table 1). Pan-tissue has been widely used and has shown that faster epigenetic aging is associated with multiple age-related diseases and conditions (e.g., Alzheimer’s, cancer, cardiovascular diseases (CVD)) indicating that epigenetic age is an indicator of health status. Some risk factors for type 2 diabetes (T2D) including BMI, waist circumference, and fasting glucose have been associated with higher Pan-tissue epigenetic age acceleration (EAA = epigenetic age − chronological age) [5, 6]. On average liver Pan-tissue EAA increased significantly by 0.33 years per BMI unit [7].

Pan-tissue performed sub-optimally estimating fibroblast age in in vitro studies. Therefore, Horvath et al. described Skin & Blood DNAm age using a similar method to Pan-tissue which performed remarkably well across a wide spectrum of cells that are most frequently used in in vitro studies, including the blood (Supplementary Table 1). Skin & Blood EAA was highly predictive of time to all-cause mortality. It was positively correlated with waist/hip ratio (WHR), blood insulin, glucose, triglyceride, systolic blood pressure, and BMI and negatively correlated with HDL and physical exercise. However, the respective correlation coefficients were weak (|*r*| < 0.11) [2].

Pan-tissue and Skin & Blood were both developed using chronological age as a surrogate for biological age. Therefore, they may not capture CpGs that signal departure of biological age from chronological age. In a newer method called PhenoAge, chronological age was replaced with a surrogate measure of phenotypic age developed using clinical data. A Cox penalized regression model was applied where the hazard of mortality was regressed on 42 clinical markers and chronological age to select variables for inclusion in the phenotypic age score. Nine clinical markers and chronological age were selected and used to estimate the 10-year mortality risk score which was then converted into units of years. Finally, the resulting phenotypic age estimate was regressed on CpG methylation levels using an elastic net regression model (Supplementary Table 1). A 1-year increase in PhenoAge was associated with a 4.5% increase in the risk of all-cause mortality in independent populations without diabetes. PhenoAge predicted mortality significantly better than Pan-tissue. It was also associated with increased risk of CVD and differed significantly between never, current, and former smokers. It was also positively correlated with blood insulin, glucose, triglyceride, and WHR and negatively correlated with HDL and physical exercise [8].

Most recently, Horvath et al. defined another DNAm age calculator called GrimAge. To develop this method, 88 plasma proteins and smoking pack-years were individually regressed on chronological age, sex, and the CpGs methylation levels using an elastic net regression model. Twelve plasma proteins and pack-years had high correlations between their DNAm estimation and the corresponding measured levels. These twelve DNAm estimated plasma proteins and smoking pack-years as well as chronological age and sex were regressed on the hazard of aging-related mortality using a Cox penalized regression model. This selected DNAm pack-years, age, sex, and the predicted DNAm for 7 of the 12 plasma proteins. The combined estimate of these factors was then transformed into GrimAge which has the same mean and variance as chronological age (Supplementary Table 1). GrimAge was highly predictive of lifespan and time-to-CVD even after adjustment for known risk factors and outperformed Pan-tissue and PhenoAge. It was also associated with hypertension and T2D. In addition, GrimAge was correlated with BMI, WHR, and physical exercise; blood insulin, glucose, HbA1c, triglyceride, and HDL; and albuminuria (all correlations were in the expected directions) [9].

Only a small proportion of CpGs are common among three of the four epigenetic ages (Supplementary Figure 1), and there is only weak/moderate correlation among them [2, 8, 9].

To our knowledge, there has been no study of DNAm age in type 1 diabetes (T1D). However, telomere length, another indicator of aging, has been investigated in T1D, and it was found to be significantly shorter in T1D compared to non-diabetic subjects [10]. Shorter telomere length has also been associated with T1D duration, pulse pressure [10], BMI [11], systolic blood pressure, all-cause mortality [12], and diabetic nephropathy [13] in subjects with T1D.

Here, we investigated DNAm age calculated by all four methods in 499 subjects with T1D from the Diabetes Control and Complications Trial/Epidemiology of Diabetes Interventions and Complications (DCCT/EDIC) study and its association with diabetic complications (CVD, nephropathy, retinopathy, and neuropathy) and their risk factors [14–16]. We also examined DNAm age in a smaller subset at two time points, 16*–*17 years apart, to investigate changes in DNAm age over time.

## Results

### Illumina whole blood EPIC data

#### Comparison of the four DNAm ages with chronological age and with each other

**Fig. 1:**
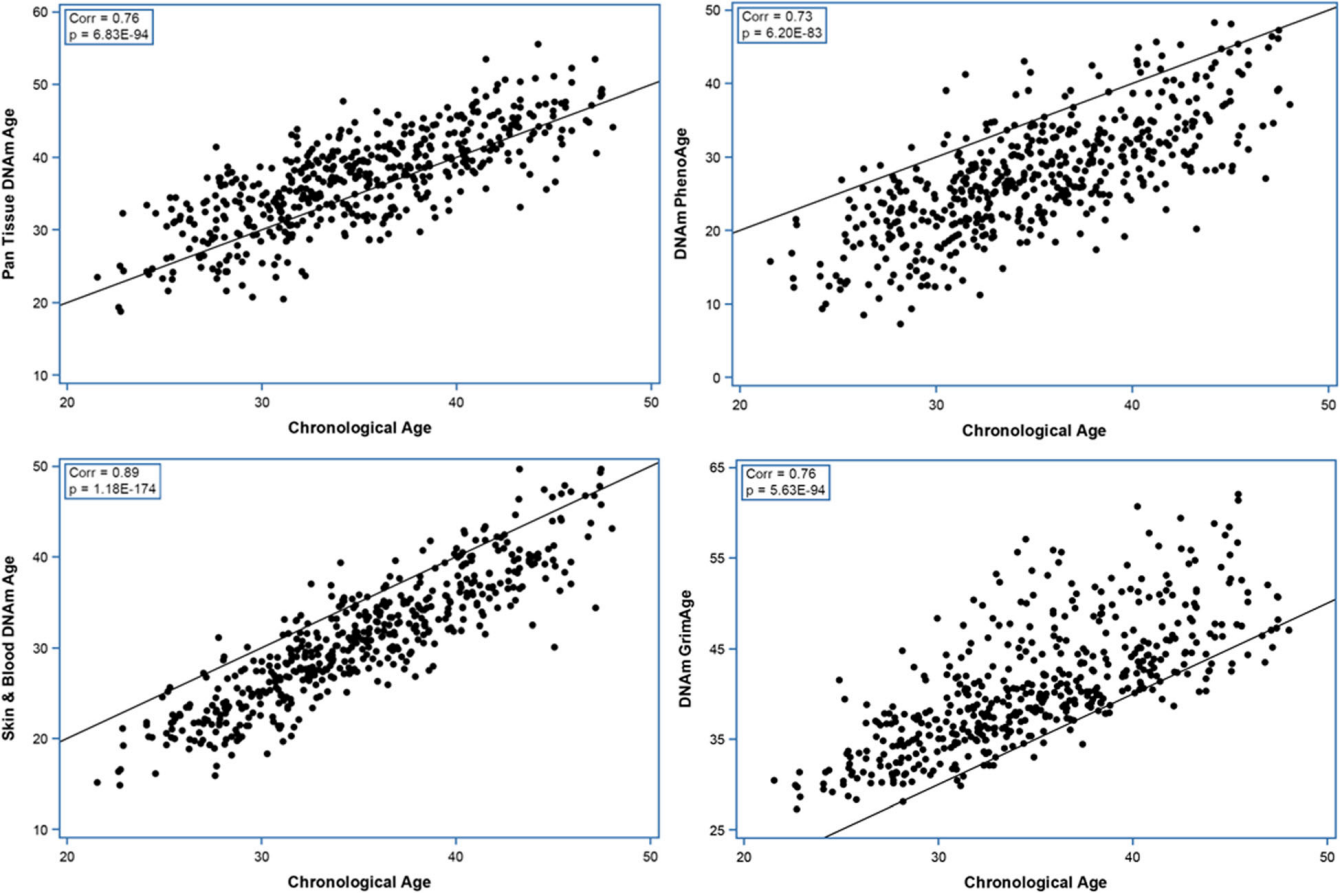
DNAm age vs. chronological age in EPIC data. Corr, Spearman correlation coefficient. The line is X = Y

Characteristics of the subjects with EPIC DNAm data are summarized in Table 1. All four epigenetic ages were highly correlated with chronological age and with each other. However, there were significant difference among them: GrimAge was higher than Pan-tissue, and both were higher than chronological age whereas Skin & Blood and PhenoAge were both lower than chronological age, and PhenoAge was lower than Skin & Blood (GrimAge > Pan-tissue > chronological age > Skin & Blood > PhenoAge) (all *p* < 0.0001) (Table 2, Fig. 1, Supplementary Figure 2).

**Table 1:** Characteristics of the subjects with Illumina EPIC array data

	Primary cohort Conventional group *N* = 124 Mean (SD)/N (%)	Primary cohort Intensive group *N* = 125 Mean (SD)/N (%)	Secondary cohort Conventional group *N* = 125 Mean (SD)/N (%)	Secondary cohort Intensive group *N* = 125 Mean (SD)/N (%)	Total *N* = 499 Mean (SD)/N (%)
Sex (male)	73 (58.9%)	60 (48.0%)	65 (52.0%)	73 (58.4%)	271 (54.3%)
Stimulated C-peptide at DCCT eligibility (pmol/ml)	0.18 (0.14)	0.17 (0.14)	0.08 (0.09)	0.06 (0.06)	0.12 (0.12)
Time-weighted HbA1c (%)*	8.93 (1.23)	6.93 (0.72)	8.73 (1.18)	7.13 (0.73)	7.92 (1.34)
Current smoker	24 (19.4%)	19 (15.2%)	25 (20.0%)	27 (21.6%)	95 (19.0%)
Alcohol consumption					
None	81 (65.3%)	80 (64.0%)	83 (66.4%)	82 (65.6%)	326 (65.3%)
Occasional	11 (8.9%)	12 (9.6%)	8 (6.4%)	11 (8.8%)	42 (8.4%)
Regular	32 (25.8%)	33 (26.4%)	34 (27.2%)	32 (25.6%)	131 (26.3%)
Physical activity at job^†^					
Sedentary	57 (46.0%)	49 (39.2%)	49 (39.2%)	55 (44.0%)	210 (42.1%)
Moderate	64 (51.6%)	69 (55.2%)	74 (59.2%)	63 (50.4%)	270 (54.1%)
Strenuous	3 (2.4%)	7 (5.6%)	2 (1.6%)	7 (5.6%)	19 (3.8%)
Leisure time physical activity^‡^					
METs < 450	38 (30.7%)	47 (37.6%)	38 (30.4%)	52 (41.6%)	175 (35.1%)
METs 450–1500	39 (31.5%)	34 (27.2%)	41 (32.8%)	34 (27.2%)	148 (29.7%)
METs > 1500	46 (37.1%)	44 (35.2%)	46 (36.8%)	39 (31.2%)	175 (35.1%)
Missing	1 (0.8%)	0 (0%)	0 (0%)	0 (0%)	1 (0.2%)
BMI (kg/m^2^)	24.9 (2.8)	25.4 (3.3)	25.0 (2.8)	26.6 (4.1)	25.5 (3.4)
Systolic blood pressure (mmHg)	112.6 (10.8)	112.3 (11.4)	116.2 (11.5)	116.3 (10.6)	114.4 (11.2)
Diastolic blood pressure (mmHg)	73.9 (8.2)	72.3 (7.7)	73.8 (8.1)	74.4 (8.2)	73.6 (8.1)
HDL (mg/dl)	52.9 (13.0)	54.9 (14.7)	51.0 (11.4)	48.8 (11.4)	51.9 (12.9)
LDL (mg/dl)	107.7 (30.2)	111.5 (27.0)	114.4 (31.0)	111.1 (28.0)	111.2 (29.1)
Triglyceride (mg/dl)	75.3 (41.1)	74.2 (45.1)	88.1 (54.2)	76.7 (39.3)	78.6 (45.5)
Total cholesterol (mg/dl)	175.7 (34.4)	181.2 (31.3)	183.0 (34.6)	175.3 (30.9)	17.8 (32.9)
Pulse rate (beat/min)	71.1 (9.8)	71.2 (9.1)	72.9 (9.3)	73.5 (9.3)	72.2 (9.4)
Age at diagnosis (years)	26.5 (5.9)	26.9 (5.4)	19.8 (7.1)	19.5 (6.7)	23.2 (7.2)
Duration (months)	88.1 (28.1)	85.7 (23.9)	173.5 (51.8)	184.8 (48.5)	133.1 (61.2)
Chronological age (years)	34.6 (6.1)	34.8 (5.6)	35.0 (5.3)	35.6 (5.8)	35.0 (5.7)
Pan-tissue					
DNAm age (years)	35.9 (6.8)	36.5 (6.3)	37.3 (6.5)	38.2 (6.5)	37.0 (6.5)
Epigenetic age acceleration (years)	1.3 (4.5)	1.8 (4.1)	2.4 (4.0)	2.5 (4.5)	2.0 (4.3)
Skin & Blood					
DNAm age (years)	30.1 (7.5)	30.2 (6.6)	30.7 (6.5)	32.1 (6.6)	30.8 (6.9)
Epigenetic age acceleration (years)	− 4.5 (3.1)	− 4.5 (3.1)	− 4.3 (3.2)	− 3.5 (3.2)	− 4.2 (3.2)
PhenoAge					
DNAm age (years)	27.1 (8.1)	26.8 (7.3)	27.0 (7.8)	28.2 (8.0)	27.3 (7.8)
Epigenetic age acceleration (years)	− 7.5 (5.8)	− 8.0 (5.0)	− 7.9 (5.0)	− 7.4 (5.7)	− 7.7 (5.4)
GrimAge					
DNAm age (years)	40.3 (6.9)	40.3 (6.6)	40.7 (6.5)	42.2 (7.0)	40.8 (6.8)
Epigenetic age acceleration (years)	5.7 (4.7)	5.5 (4.3)	5.7 (4.8)	6.6 (5.0)	5.9 (4.7)

All factors were obtained at DNAm measurement except for stimulated C-peptide which is measured at DCCT eligibility

*MET* metabolic equivalent of task

*Time-weighted HbA1c since DCCT baseline

†Level of activity on the job, at school, or in home making: sedentary such as office work with occasional inter-office walking; moderate activity requires considerable but not constant lifting, walking, bending, pulling, etc. such as homemaker with family and without domestic assistance; and strenuous activity requires almost constant lifting, bending, pulling, scrubbing, etc. such as furniture mover

‡According to the international classification by Ainsworth used by American College of Sports Medicine (ACSM), light, moderate, hard, and very hard activity was allocated 3, 4, 6, and 9 METs, respectively. For each participant, these allocated MET values were multiplied by the time (min) spent in that activity to obtain the MET for that level of activity. The sum of METs from all activities was recorded as the total leisure time activity for each participant. Subjects then were categorized into three groups based on the ACSM recommendation for METs min/week [17, 18]

**Table 2:** Correlation and mean difference between epigenetic ages and chronological age in EPIC data

	Chronological Age	Pan Tissue	Skin & Blood	PhenoAge	GrimAge
Chronological Age	-	0.76	0.89	0.73	0.76
Pan Tissue	2.0 (4.3)	-	0.75	0.73	0.57
Skin & Blood	-4.2 (3.1)	-6.2 (4.8)	-	0.72	0.72
PhenoAge	-7.7 (5.4)	-9.7 (5.4)	-3.5 (5.5)	-	0.64
GrimAge	5.9 (4.7)	3.9 (6.2)	10.1 (5.3)	13.6 (6.2)	-

The values above the diagonal are Spearman correlation coefficients. The values below the diagonal are mean differences (mean of the age in the row − mean of the age in the column) and their corresponding SDs in brackets. All *p* values regarding the correlations and the mean differences are < 0.0001

#### Changes in EAA by chronological age

**Fig. 2:**
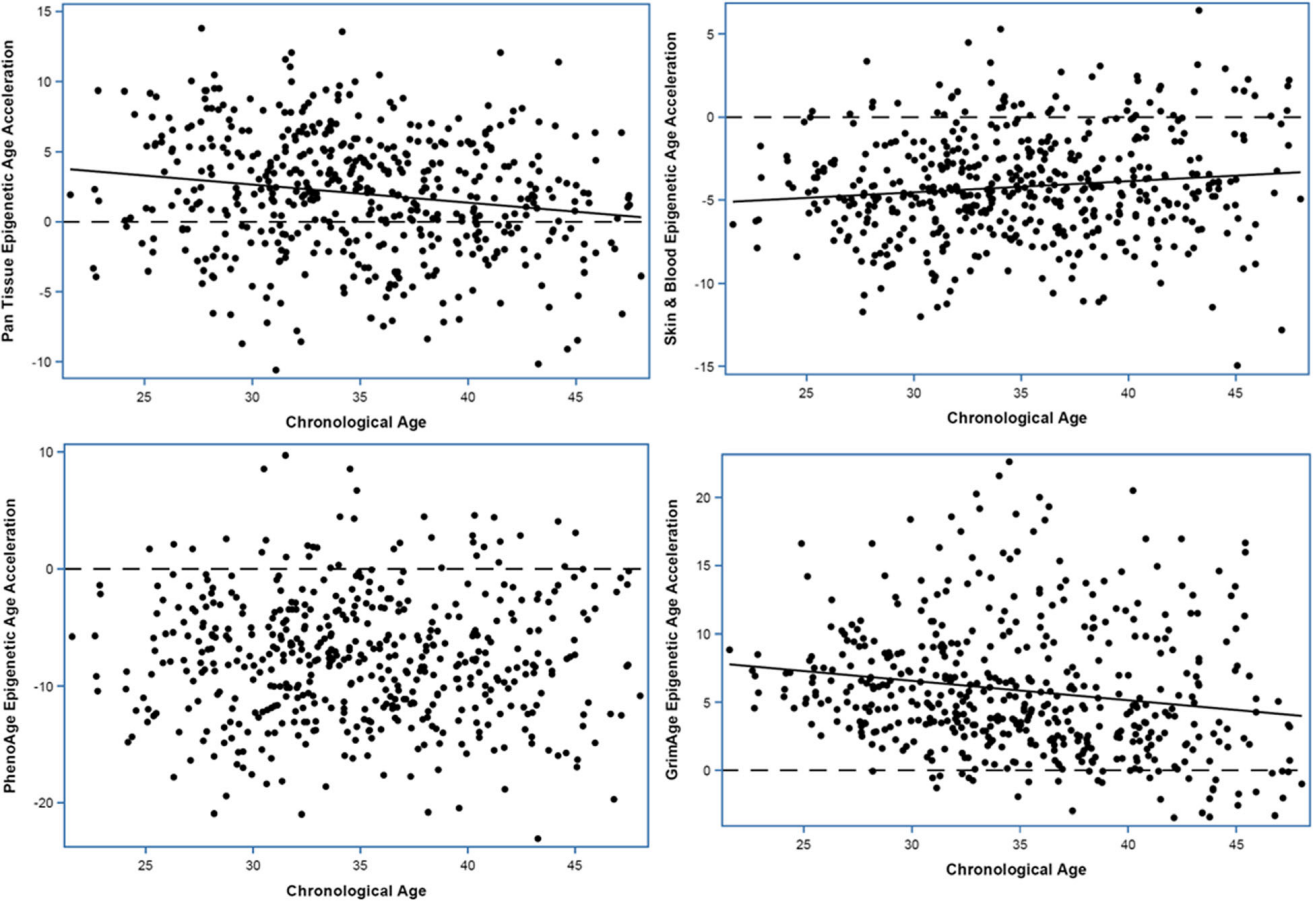
Epigenetic age acceleration vs. chronological age in EPIC data. The dash line is epigenetic age acceleration = 0. The solid line is the line fitting linear regression model with chronological age as predictor and epigenetic age acceleration as outcome and is present when chronological age is significantly associated with epigenetic age acceleration (*p* < 0.05)

The differences between chronological age and each of Pan-tissue, Skin & Blood, and GrimAge decreased by 0.13, 0.07, and 0.14 years per 1-year increase in chronological age (*p* = 1.18E−4, 6.49E−3, and 1.03E−4, respectively). The difference between chronological age and PhenoAge did not differ significantly by chronological age (Fig. 2, Supplementary Table 2).

#### Association of the four DNAm ages with development of diabetic complications

Epigenetic ages were not significantly associated with the development of CVD or retinopathy. Although DNAm ages were not associated with estimated glomerular filtration (eGFR), both PhenoAge (*β* = 0.023, *p* = 0.007) and GrimAge (*β* = 0.029, *p* = 0.002) were positively associated with repeated measures of albumin excretion rate (AER, natural log transformed) which remained significant after adjustment of HbA1c levels. The effect of GrimAge on AER increased over time (GrimAge × EDIC year interaction β (SE) = 0.0013 (0.0005), *p* = 5.10E−3) whereas effect of PhenoAge was not significantly different over time (PhenoAge × EDIC year interaction *p* = 0.85) (Table 3, Supplementary Figure 3-4). Pan-tissue, Skin & Blood, and PhenoAge were not associated with neuropathy, but GrimAge was positively associated with both diabetic peripheral neuropathy (DPN: OR = 1.07, *p* = 9.24E−3) and cardiovascular autonomic neuropathy (CAN: OR = 1.06, *p* = 0.011). These associations also remained significant after further adjustment for time-weighted HbA1c (Table 3).

**Table 3:** Association of four DNAm ages with development of T1D complication

	Total *N*	Events *N*	Model 1		Model 2	
CVD						
CVD events from DNAm measurement to EDIC year 20	496	58	HR (95% CI)	*p*	HR (95% CI)	*p*
Pan-tissue			1.03 (0.96–1.10)	0.433	1.03 (0.96–1.11)	0.371
Skin & Blood			1.06 (0.97–1.16)	0.207	1.07 (0.97–1.18)	0.166
PhenoAge			1.05 (0.99–1.10)	0.086	1.05 (1.00–1.11)	0.061
GrimAge			1.04 (0.99–1.10)	0.163	1.04 (0.99–1.10)	0.085
Nephropathy and renal function						
Repeated eGFR from EDIC year 0 to 18 (ml/min/1.73 m^2^)	498	NA	Beta (SE)	*p*	Beta (SE)	*p*
Pan-tissue			− 0.17 (0.10)	0.075	− 0.18 (0.10)	0.063
Skin & Blood			− 0.20 (0.13)	0.119	− 0.23 (0.13)	0.084
PhenoAge			− 0.13 (0.08)	0.099	− 0.14 (0.08)	0.068
GrimAge			0.05 (0.09)	0.531	0.03 (0.09)	0.736
Sustained eGFR <60 ml/min/1.73 m^2^ during EDIC year 0-18	498	23	HR (95% CI)	*p*	HR (95% CI)	*p*
Pan-tissue			1.11 (0.99–1.26)	0.077	1.12 (0.99–1.27)	0.075
Skin & Blood			1.08 (0.94–1.23)	0.287	1.07 (0.94–1.23)	0.318
PhenoAge			1.06 (0.98–1.15)	0.160	1.06 (0.97–1.14)	0.199
GrimAge			1.04 (0.96–1.13)	0.315	1.04 (0.95–1.13)	0.432
Repeated AER from EDIC year 0 to 18 (mg/24 h log_e_ transformed)	499	NA	Beta (SE)	*p*	Beta (SE)	*p*
Pan-tissue			0.011 (0.010)	0.273	0.011 (0.011)	0.282
Skin & Blood			0.021 (0.014)	0.146	0.021 (0.014)	0.138
PhenoAge			0.023 (0.008)	6.78E−3	0.024 (0.009)	4.83E−3
GrimAge			0.029 (0.009)	2.13E−3	0.032 (0.009)	7.23E−4
AER ≥ 300 mg/24 h during EDIC year 0–18	484	29	HR (95% CI)	*p*	HR (95% CI)	*p*
Pan-tissue			1.04 (0.94–1.15)	0.465	1.01 (0.91–1.12)	0.846
Skin & Blood			1.05 (0.92–1.19)	0.515	1.08 (0.94–1.24)	0.292
PhenoAge			1.05 (0.97–1.12)	0.211	1.04 (0.97–1.12)	0.277
GrimAge			1.06 (0.98–1.14)	0.152	1.04 (0.96–1.13)	0.309
Retinopathy						
SNPDR during EDIC year 0–18	473	92	HR (95% CI)	*p*	HR (95% CI)	*p*
Pan-tissue			1.02 (0.97–1.08)	0.396	1.01 (0.95–1.07)	0.765
Skin & Blood			1.06 (0.98–1.14)	0.136	1.04 (0.96–1.13)	0.343
PhenoAge			1.04 (0.99–1.08)	0.111	1.03 (0.98–1.07)	0.287
GrimAge			1.04 (0.99–1.08)	0.135	1.00 (0.95–1.05)	0.892
PDR during EDIC year 0–18	482	96	HR (95% CI)	*p*	HR (95% CI)	*p*
Pan-tissue			1.01 (0.96–1.07)	0.584	0.99 (0.94–1.05)	0.790
Skin & Blood			1.05 (0.98–1.14)	0.180	1.03 (0.95–1.12)	0.497
PhenoAge			1.03 (0.99–1.08)	0.131	1.02 (0.98–1.07)	0.400
GrimAge			1.03 (0.98–1.07)	0.256	0.99 (0.94–1.04)	0.698
CSME during EDIC year 0–18	464	108	HR (95% CI)	*p*	HR (95% CI)	*p*
Pan-tissue			1.04 (0.99–1.09)	0.176	1.04 (0.98–1.09)	0.179
Skin & Blood			1.00 (0.94–1.08)	0.935	0.99 (0.92–1.07)	0.827
PhenoAge			1.04 (1.00–1.08)	0.091	1.01 (0.97–1.06)	0.517
GrimAge			1.02 (0.97–1.06)	0.428	1.00 (0.95–1.04)	0.843
Neuropathy						
DPN at EDIC year 13–15	431	109	OR (95% CI)	*p*	OR (95% CI)	*p*
Pan-tissue			1.02 (0.96–1.08)	0.562	1.02 (0.96–1.09)	0.466
Skin & Blood			0.96 (0.88–1.05)	0.345	0.96 (0.88–1.05)	0.352
PhenoAge			1.02 (0.98–1.07)	0.356	1.02 (0.97–1.07)	0.448
GrimAge			1.07 (1.02–1.12)	9.24E−3	1.07 (1.01–1.12)	2.17E−2
CAN at EDIC year 13–18	446	176	OR (95% CI)	*p*	OR (95% CI)	*p*
Pan-tissue			1.01 (0.96–1.07)	0.635	1.02 (0.97–1.07)	0.526
Skin & Blood			0.99 (0.92–1.06)	0.758	0.99 (0.92–1.07)	0.844
PhenoAge			1.01 (0.97–1.05)	0.689	1.00 (0.96–1.05)	0.971
GrimAge			1.06 (1.01–1.11)	1.07E−2	1.06 (1.01–1.11)	2.39E−2

Model 1, adjusted for batch, cell proportions, sex, age, and T1D duration at DNAm measurement; model 2, adjusted for all covariates in model 1 plus repeated measures of HbA1c

*HR* hazard ratio, *OR* odds ratio, *CI* confidence interval, *SE* standard error, *CVD* cardiovascular diseases, *eGFR* estimated glomerular filtration rate, *AER* albumin excretion rate, *SNPDR* sever non-proliferative diabetic retinopathy, *PDR* proliferative diabetic retinopathy, *CSME* clinically significant macular edema, *DPN* diabetic peripheral neuropathy, *CAN* cardiovascular autonomic neuropathy

#### Association of risk factors of diabetic complication with the four DNAm ages

The univariable associations of different factors with EAAs are shown in Supplementary Table 2. The results regarding multivariable associations of only sex, age, T1D duration, and time-weighted HbA1c with epigenetic ages (minimal model) are shown in Supplementary Table 3. In the multivariable analysis when all factors were included, males had on average 1.5 years higher Pan-tissue (*p* = 8.00E−4) and GrimAge (*p* = 9.99E−5) compared to females whereas females had on average 1.5 years higher PhenoAge compared to males (*p* = 0.005) (Table 4). PhenoAge increased 0.4 years per 1% increase in time-weighted HbA1c (*p* = 0.026) and 0.01 years per 1-month increase in T1D duration (*p* = 0.043) (Table 4). The effect of time-weighted HbA1c on PhenoAge was not significantly different in either conventional (*β* (SD) = 0.45 (0.29), *p* = 0.12) or intensive (*β* (SD) = 0.15 (0.48), *p* = 0.76) therapy group (interaction *p* = 0.56). Skin & Blood increased 0.09 years per one-unit increase in BMI (*p* = 0.048) (Table 4). Those with strenuous physical activity at work on average had 1.99 years lower Pan-tissue compared to those with sedentary jobs (*p* = 0.045), and those who achieved one to two times the recommended level of physical activity during leisure time on average had 0.8 years lower Skin & Blood compared to those who did not achieve the recommended level (*p* = 0.022) (Table 4). Current smokers had on average 7.1 years higher GrimAge compared to non-smokers (*p* = 9.03E−50). The other factors were not significantly associated with epigenetic ages (Table 4).

**Table 4:** Multivariable association of different factors with DNAm ages in the EPIC dataset

	Pan-tissue		Skin & Blood		PhenoAge		GrimAge	
Factor	Beta (SE)	*p*	Beta (SE)	*p*	Beta (SE)	*p*	Beta (SE)	*p*
Sex (male)	1.47 (0.44)	8.00E−4	− 0.42 (0.32)	0.18	− 1.50 (0.53)	4.57E−3	1.45 (0.37)	9.99E−5
Cohort (primary)	− 0.27 (0.58)	0.64	− 0.14 (0.42)	0.74	0.82 (0.69)	0.24	− 0.71 (0.49)	0.15
Age (years)	0.85 (0.03)	1.16E−86	1.06 (0.02)	3.48E−160	0.94 (0.04)	2.39E−77	0.80 (0.03)	2.21E−99
T1D Duration (months)	0.009 (0.005)	0.06	0.003 (0.004)	0.43	0.01 (0.01)	4.29E−2	− 0.005 (0.004)	0.24
Stimulated C-peptide (pmol/ml)	− 0.15 (1.79)	0.93	− 0.72 (1.30)	0.58	− 1.74 (2.15)	0.42	− 2.05 (1.51)	0.18
Time-weighted HbA1c (%)*	0.05 (0.14)	0.71	− 0.01 (0.10)	0.89	0.38 (0.17)	2.57E−2	0.02 (0.12)	0.86
BMI (kg/m2)	0.02 (0.06)	0.76	0.09 (0.04)	4.84E−2	0.09 (0.07)	0.20	− 0.02 (0.05)	0.72
Systolic blood pressure (mmHg)	− 0.01 (0.02)	0.66	0.004 (0.017)	0.79	− 0.03 (0.03)	0.33	− 0.01 (0.02)	0.48
Diastolic blood pressure (mmHg)	0.01 (0.03)	0.75	− 0.01 (0.02)	0.53	0.005 (0.038)	0.90	0.01 (0.03)	0.83
HDL (mg/dl)	0.93 (0.65)	0.15	0.01 (0.47)	0.98	0.59 (0.78)	0.45	− 0.11 (0.55)	0.85
LDL (mg/dl)	0.91 (0.65)	0.16	0.02 (0.47)	0.96	0.60 (0.78)	0.45	− 0.09 (0.55)	0.87
Triglyceride (mg/dl)	0.18 (0.13)	0.16	0.001 (0.094)	0.99	0.11 (0.16)	0.48	− 0.02 (0.11)	0.85
Total cholesterol (mg/dl)	− 0.91 (0.65)	0.16	− 0.02 (0.47)	0.97	− 0.58 (0.78)	0.46	0.10 (0.55)	0.85
Pulse rate (beat/min)	− 0.01 (0.02)	0.65	0.01 (0.02)	0.38	0.01 (0.03)	0.75	0.01 (0.02)	0.67
Current smoker vs. non-smoker	− 0.16 (0.50)	0.76	− 0.51 (0.36)	0.16	0.99 (0.60)	0.10	7.13 (0.42)	9.03E−50
Regular drinker vs. non-drinker	− 0.37 (0.46)	0.42	0.26 (0.33)	0.43	0.38 (0.55)	0.49	0.69 (0.38)	0.07
Occasional drinker vs. non-drinker	− 0.66 (0.67)	0.32	− 0.50 (0.49)	0.30	0.41 (0.81)	0.61	0.71 (0.57)	0.21
Strenuous activity vs. sedentary^†^	− 1.99 (0.99)	4.53E−2	− 0.85 (0.72)	0.24	− 0.94 (1.20)	0.43	0.54 (0.84)	0.52
Moderate activity vs. sedentary^†^	− 0.11 (0.39)	0.78	0.23 (0.28)	0.41	0.05 (0.47)	0.91	0.25 (0.33)	0.44
METS > 1500 vs. METs < 450^‡^	− 0.18 (0.47)	0.71	− 0.27 (0.34)	0.42	0.60 (0.57)	0.29	− 0.33 (0.40)	0.41
METs 450–1500 vs. METs < 450^‡^	− 0.56 (0.48)	0.25	− 0.81 (0.35)	2.15E−2	− 0.56 (0.58)	0.34	− 0.76 (0.41)	0.06

Cell counts (B, CD4T, CD8T, natural killer, eosinophil, and monocyte) and batch (as a categorical variable) were also included in the multivariable analysis. All factors were obtained at DNAm measurement except for stimulated C-peptide which is measured at DCCT eligibility

*MET* metabolic equivalent of task

*Time-weighted HbA1c from DCCT baseline to DNAm measurement

^**†**^Level of activity on the job, at school, or in home making: sedentary such as office work with occasional inter-office walking; moderate activity requires considerable but not constant lifting, walking, bending, pulling, etc. such as homemaker with family and without domestic assistance; and strenuous activity requires almost constant lifting, bending, pulling, scrubbing, etc. such as furniture mover

‡According to the international classification by Ainsworth used by American College of Sports Medicine (ACSM), light, moderate, hard, and very hard activity was allocated 3, 4, 6, and 9 METs, respectively. For each participant, these allocated MET values were multiplied by the time (min) spent in that activity to obtain the MET for that level of activity. The sum of METs from all activities was recorded as the total leisure time activity for each participant. Subjects then were categorized into three groups based on the ACSM recommendation for METs min/week [17, 18]

### Illumina 450K whole blood data

Characteristics of the subjects with 450K data are summarized in Supplementary Table 4. Chronological age and all four epigenetic ages were highly correlated. However, there were significant differences among them: Pan-tissue > (greater than) GrimAge > Skin & Blood > chronological age > PhenoAge (all *p* < 0.0001) (Supplementary Table 5, Supplementary Figure 5-6). EAAs were not significantly different in the two treatment groups (Supplementary Table 6).

The difference between chronological age and GrimAge decreased by 0.13 years per 1-year increase in chronological age (*p* = 0.01) (Supplementary Table 6 and Figure 9).

Sex, cohort, treatment group, T1D duration, stimulated C-peptide, and time-weighted HbA1c were not significantly associated with epigenetic ages (Supplementary Table 6, Table 5).

**Table 5:** Multivariable association of different factors with DNAm ages in 450K dataset

	Pan-tissue		Skin & Blood		DNAm		DNAm	
Factor	Beta (SE)	*p*	Beta (SE)	*p*	Beta (SE)	*p*	Beta (SE)	*p*
Whole blood								
Sex (male)	− 0.19 (1.22)	0.88	− 1.54 (0.88)	0.09	− 2.08 (1.66)	0.22	1.43 (0.77)	0.07
Cohort (primary)	0.93 (1.71)	0.59	0.33 (1.23)	0.79	− 0.15 (2.33)	0.95	0.04 (1.07)	0.97
Group (intensive)	1.25 (3.89)	0.75	2.67 (2.81)	0.35	1.42 (5.30)	0.79	3.40 (2.44)	0.17
Age (years)	1.08 (0.09)	4.76E−16	1.03 (0.07)	1.13E−20	1.05 (0.12)	2.96E−11	0.84 (0.06)	1.92E−19
T1D Duration (months)	0.015 (0.014)	0.29	0.009 (0.010)	0.37	0.002 (0.019)	0.9	0.006 (0.009)	0.49
Stimulated C-peptide (pmol/ml)*	− 5.11 (5.97)	0.40	− 1.56 (4.31)	0.72	− 4.78 (8.12)	0.56	4.56 (3.75)	0.23
Time-weighted HbA1c (%)†	0.70 (0.98)	0.48	0.64 (0.71)	0.37	0.58 (1.34)	0.67	1.08 (0.62)	0.09
Monocyte								
Sex (male)	2.61 (1)	1.18E−2	− 0.51 (0.91)	0.58	− 2.64 (1.62)	0.11	1.3 (0.81)	0.11
Cohort (primary)	− 1.2 (1.49)	0.42	− 0.95 (1.36)	0.49	− 0.98 (2.42)	0.69	0.21 (1.2)	0.86
Group (intensive)	− 0.45 (1.87)	0.81	1.36 (1.7)	0.43	2.58 (3.03)	0.39	0.94 (1.5)	0.53
Age (years)	− 0.21 (0.08)	1.16E−2	− 0.2 (0.07)	9.46E−3	0.92 (0.13)	5.50E−09	− 0.20 (0.07)	4.18E−3
T1D Duration (months)	− 0.01 (0.01)	0.32	− 0.01 (0.01)	0.29	− 0.02 (0.02)	0.31	− 0.01 (0.01)	0.49
Stimulated C-peptide (pmol/ml)*	− 13.05 (5.25)	1.62E−2	− 5.59 (4.78)	0.25	− 14.48 (8.51)	0.09	1.07 (4.23)	0.80
Time-weighted HbA1c (%)†	0.55 (0.71)	0.45	0.45 (0.65)	0.49	0.59 (1.15)	0.61	0.55 (0.57)	0.34

Cell counts (B, CD4T, CD8T, natural killer, eosinophil, and monocyte) were also included in the multivariable whole blood analysis

*Stimulated C-peptide at DCCT eligibility

†Time-weighted HbA1c from DCCT baseline to monocyte DNAm measurement

### Illumina 450K monocyte data

Chronological age and all four epigenetic ages were highly correlated (Supplementary Table 7, Supplementary Figure 8-9). However, except for Pan-tissue and GrimAge, there were significant differences among them: PhenoAge > Pan-tissue ≈ GrimAge > Skin & Blood > chronological age (*p* < 0.001).

Although Pan-tissue EAA was on average 2.9 years lower in the former DCCT intensive versus conventional treatment group, the difference was not significant in the multivariable analysis. The other EAAs were also not significantly different between the two treatment groups (Table 5, Supplementary Table 8).

The differences between chronological age and each of Pan-tissue, Skin & Blood, and GrimAge decreased by 0.3, 0.2, and 0.2 years per 1-year increase in chronological age (*p* = 0.005, 0.003, and 0.004, respectively) (Supplementary Table 8 and Figure 10).

**Fig. 3:**
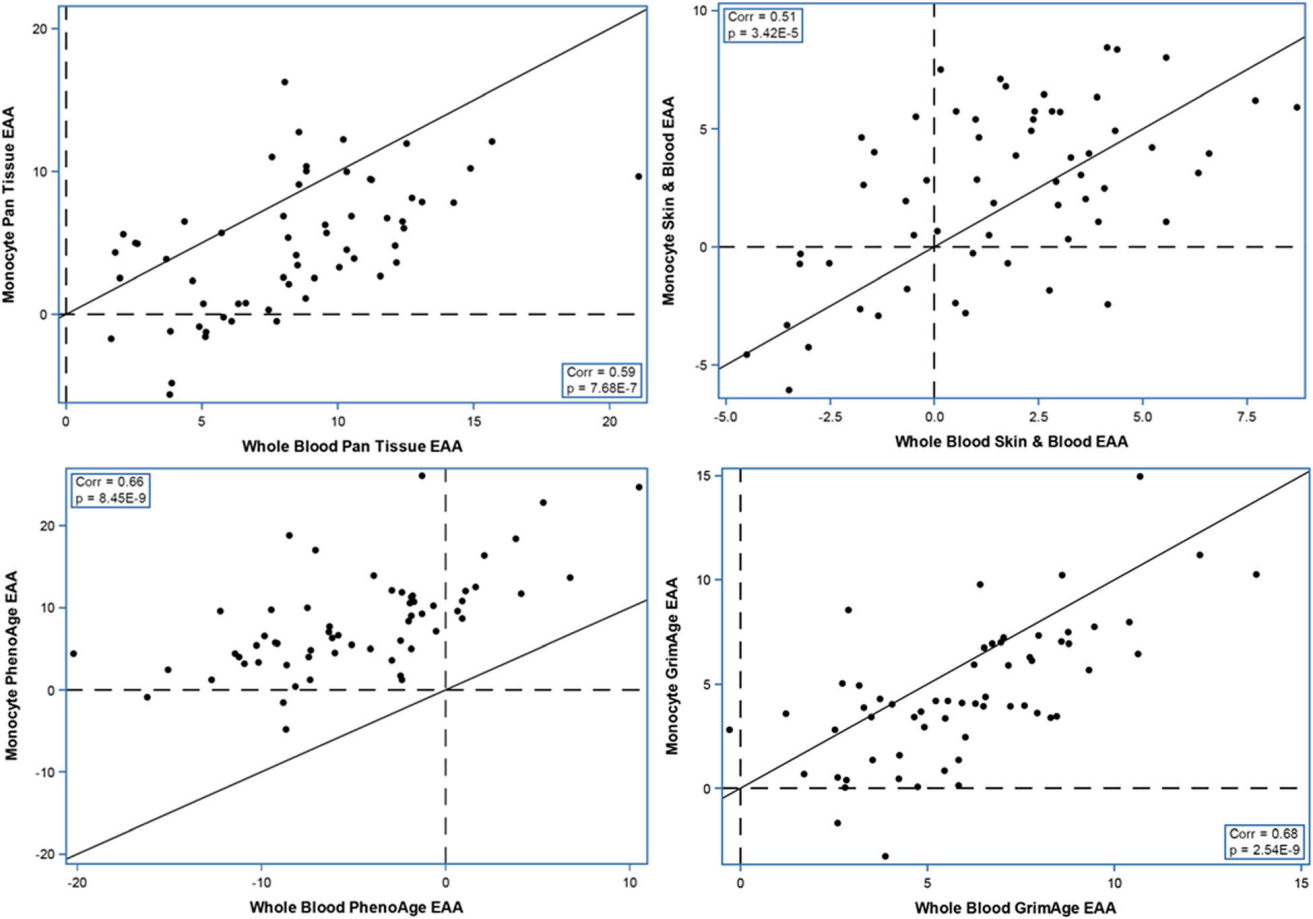
Monocyte vs. whole blood epigenetic age acceleration in 450K data. EAA, epigenetic age acceleration; Corr, Spearman correlation coefficient. The solid line is X = Y. The dash lines are EAA = 0

All four EAAs were highly correlated between whole blood and monocyte (Fig. 3).

In the multivariable analysis, Pan-tissue on average decreased 13.05 years per 1 pmol/ml increase in stimulated C-peptide at DCCT eligibility (*p* = 0.016). Association of C-peptide with Pan-tissue was not significantly different in the two treatment groups (*p* = 0.61). Sex, cohort, treatment group, T1D duration, and time-weighted HbA1c were not significantly associated with epigenetic ages (Table 5).

## Discussion

We used DNAm data in whole blood measured by Illumina EPIC array and four different methods to estimate epigenetic ages in 499 subjects with T1D. Subsequently, we compared estimated epigenetic ages with chronological age and investigated if the epigenetic ages were associated with development of T1D complications (CVD, nephropathy/decreased renal function, retinopathy, and neuropathy) and their risk factors.

All four epigenetic ages were correlated with chronological age and with each other, but there were significant differences between them. Pan-tissue and Skin & Blood were developed to predict chronological age in healthy individuals whereas PhenoAge and GrimAge used biological biomarkers associated with time-to-death to predict differences in life expectancy of individuals. In addition, they were developed and tested in datasets measured in different tissues by different arrays (Illumina 27K, 450K, and EPIC) and used different statistical methods. As a result, there have been only low to moderate correlations between them [2, 8, 9]. Pan-tissue and GrimAge were significantly higher than chronological age, but Skin & Blood and PhenoAge were significantly lower than chronological age. T1D usually has a negative impact on general health [19, 20]. Therefore, we expected DNAm age to be higher than chronological age in subjects with T1D [21]. However, DCCT subjects were a relatively healthy group of subjects with T1D at baseline due to the extensive inclusion/exclusion criteria applied. Out of ~ 7000 individuals who made initial contact, only 1441 subjects aged 13–39 years with 1–15 years of T1D and no serious long-term complications of diabetes were included in DCCT. Subjects were excluded if they were at risk for adverse effects (e.g., history of frequent ketoacidosis, hypoglycemic coma, or seizure) had known risk factors for vascular complications, were unlikely to comply with the demands of treatment protocols, did not demonstrate an adequate understanding of the DCCT’s purpose, or had drug addiction, chronic alcoholism, or major mental illness [22]. In addition, individuals with EPIC DNAm data are not an entirely random sample of DCCT/EDIC subjects (Supplementary Tables 9-13).

With the exception of PhenoAge, the difference between the epigenetic ages and chronological age decreased in older subjects. This is consistent with previous findings where longitudinal changes in Pan-tissue EAA were tracked using linear mixed models (LMMs) within five different cohorts and showed that epigenetic age increases at a slower rate than chronological age across the life span especially in older populations [23].

We did not find significant association between epigenetic ages and development of CVD. Studies have investigated the association of Pan-tissue and risk of developing CVD in non-diabetic subjects. In 2543 African Americans, the hazard ratio of fatal coronary heart disease increased by 1.03 per year increase in Pan-tissue over a ≈ 17-year follow-up period (144 events) [21]. Similarly, in a cohort of 1863 subjects aged 50–75 years from Germany, the risk of CVD mortality increased by 1.04 per year increase in Pan-tissue over 13 years of follow-up (194 events) [24]. However, our study had only 22% power to detect an effect with this size, due to the relatively small number (*N* = 58) of CVD events. Another study did not find any significant association between Pan-tissue and incidence of CVD in women from African, Caucasian, and Hispanic ancestry [25]. This discrepancy could be due to race, gender, age, etc. differences between the studied populations as well as statistical power. Increases in PhenoAge and GrimAge have also been reported in association with increased risk of CVD in non-diabetic subjects (*β* = 1.10 and HR = 1.07, respectively) [8, 9]. However, our study was again under-powered to detect these effects (power = 0.55 and 0.72, respectively). It is noteworthy that in all these studies including the current study, Pan-tissue was calculated in whole blood which is not the target tissue for diabetic complication. Although it has been shown that DNAm profiles are quite similar in different tissues, there are tissue-specific differentially methylated regions which can affect DNAm calculation [26]. However, accessing target tissues are not feasible especially in large scale studies of living subjects.

To our knowledge, association of DNAm age with risk of developing nephropathy, retinopathy, or neuropathy has not been investigated before. We did not observe any significant association between DNAm age and development of nephropathy and retinopathy. However, we found higher GrimAge and PhenoAge to be associated with higher levels of repeated measures of AER, an indicator of decreasing renal function. These associations remained significant even after adjusting for time-weighed HbA1c indicating that at least part of the effects is independent of HbA1c levels. This result is consistent with previous findings where GrimAge has been associated with albuminuria in non-diabetic subjects [9]. Of the four epigenetic ages, higher GrimAge was also associated with development of both DPN and CAN. Larger number of events and higher statistical power along with relatively larger effect sizes could be among the reasons that we detected association of GrimeAge with neuropathy but not the rest of the complications, although CSME has similar number of cases.

Intensive therapy and keeping HbA1c levels close to the normal range has been associated with decreased risk of developing diabetic complications [27]. In our study, both time-weighted HbA1c (but not treatment group) and T1D duration were associated with higher PhenoAge but not with the other epigenetic ages.

Males had higher Pan-tissue and GrimAge compared to females consistent with previous findings [9, 21, 25] whereas PhenoAge was higher in women and sex was not associated with Skin & Blood. All Pan-tissue and PhenoAge CpGs are on autosomal chromosome. Only one of Skin & Blood CpG (cg01892695) is on the X chromosome. Therefore, sex differences in Pan-tissue and PhenoAge are not due to CpGs being on the X chromosome. The CpGs included in GrimAge are not publicly available.

The majority of known risk factors for diabetic complications were not associated with the four epigenetic ages. Two studies have investigated the association of CVD risk factors with Pan-tissue in non-diabetic subjects, but they reported conflicting results [21, 25]. In our study, individuals with physically strenuous jobs had significantly lower Pan-tissue compared to individuals with sedentary jobs, and those who achieved one to two times the recommended level of physical activity during leisure time had lower Skin & Blood compared to those who did not achieve the recommended level. Consistent with these findings, a twin study found that although only a small amount of variance in Pan-tissue is explained by non-shared environmental factors in younger individuals, leisure time physical activity can affect Pan-tissue during adult years [28]. Higher BMI was associated with higher Skin & Blood consistent with what has been observed in non-diabetic subjects before [2]. Current smoking had a large effect on GrimAge; GrimAge increased on average 7 years in current smokers compared to non-smokers. This finding was expected since pack-years were one of the surrogate biomarkers used to generate GrimAge [9]. Triglyceride, HDL, BMI, and physical exercise have been correlated with PhenoAge and/or GrimAge [8, 9]. In our study, although some of these factors were associated with PhenoAge and/or GrimAge in the univariable analysis, these associations did not remain significant in the multivariable analysis [22].

We also investigated epigenetic age in a smaller subset (*N* = 63) using Illumina 450K array in whole blood and 16–17 years later in monocytes which gave us the opportunity to investigate the difference between the two arrays and the change in epigenetic age over time. In 450K whole blood data, on average, Pan-tissue had the highest estimated value whereas in EPIC data GrimAge was estimated higher than the other epigenetic ages. In addition, Skin & Blood, which was on average lower than chronological age in EPIC data, was on average higher than chronological age in 450K data. Therefore, it appears that the type of array can affect the estimated epigenetic age. However, this could also be due to subjects being highly selected (two extremes of HbA1c and complications risk) in 450K data. Epigenetic ages in whole blood and monocyte measured 16–17 years apart were always correlated indicating that DNAm age is consistent over time and in multiple cell types. This is consistent with previous findings showing that Pan-tissue is consistent across life span [23], and a substantial amount (over 70%) of its changes are shared between different tissue/cell types [29] and also PhenoAge being correlated in different tissues and cell types [8]. In monocyte 450K data, we found a new association which we did not observe in EPIC data: higher stimulated serum C-peptide at DCCT eligibility was associated with lower monocyte Pan-tissue measured decades later at EDIC year 16–17. The association was in the expected direction since preserved beta cell function as measured by stimulated C-peptide is associated with better clinical outcomes (i.e., better glycemic control [30] and lower risk for diabetic complications [31–34]). The fact that this association was not observed in EPIC data with a much larger sample size could partly be due to the fact that the two sample populations are different: the 450K sample was selected from two extremes of HbA1c and complications risk, whereas the EPIC sample was selected randomly from each cohort/treatment group. In addition, probes may perform slightly differently between the two arrays (450K and EPIC), and as a result measured levels of methylation can be different [35]. In addition, whole blood is a mixture of different cells with different half-lives which could dilute the association(s) of individual cell types especially if they make up only a small proportion (e.g., monocytes, 2–8%).

We investigated the physical distance between all CpGs that are included in Pan-tissue, Skin & Blood, and PhenoAge epigenetic age calculations and T1D GWAS loci [36, 37]: all CpGs are > 25 Mb away from them. Therefore, it is unlikely that estimated DNAm ages were confounded by methylation levels of the CpGs associated with T1D. However, the CpGs included in GrimAge are not publicly available. There have been two epigenome-wide association studies of T1D [38, 39]; however, associated CpGs (*N* = 132) are available for one of them [38]. Of these, only two CpGs are common with Pan-tissue (cg02047577 (Chr20, 62,587,702 (HG19)) and cg16494477 (Chr5, 170,847,251)): the later CpG and another site are in common with PhenoAge (cg11903057 (Chr4, 40,198,776) and cg16494477 (Chr5, 170,847,251)). Therefore, it appears unlikely that they can have major effect on DNAm age.

## Conclusions

We found that although all four epigenetic ages are correlated with each other and with chronological age, however, there are significant differences between them in subjects with T1D. We also found that DNAm age is consistent over time, but type of array (450K vs. EPIC) and cell type can affect the estimated epigenetic age. None of the epigenetic ages were associated with CVD or retinopathy, but PhenoAge and GrimAge were both associated with decreasing renal function as measured by AER. GrimAge was also associated with both DPN and CAN. Only PhenoAge was positively associated with HbA1c levels and T1D duration, two major risk factors for diabetic complications. Some of the other risk factors of diabetic complications were associated with individual epigenetic ages. Therefore, it seems that the investigated epigenetic ages all work sub-optimally in detecting subjects with T1D who are at higher risk to develop complications. However, PhenoAge and specifically GrimAge performed better that Pan-tissue and Skin & Blood suggesting that including biomarkers associated with aging-related mortality improves the accuracy of DNAm measurement. Nevertheless, only some of the risk factors of diabetic complication which are also among the main factors associated with aging-related mortality in the general population were considered in their development (serum creatinine and glucose in PhenoAge and smoking pack-years in GrimAge), and major factors such as hypertension, lipid levels, BMI, and HbA1c which is a better indicator of glycemic levels in long-term compared to serum glucose were not considered [8, 9]. Including these factors could potentially improve epigenetic age estimation in the general population and specifically in subjects with T1D.

## Methods

### Subjects

The study subjects were from the DCCT/EDIC study. Subjects with T1D aged 13–39 years were recruited into DCCT in 1983–1989 in two cohorts. The primary prevention cohort included participants with 1–5 years of diabetes and no pre-existing retinopathy or nephropathy. The secondary cohort included participants with 1–15 years of diabetes and pre-existing mild retinopathy. Subjects were randomly assigned to receive intensive or conventional diabetes therapy [40]. The DCCT ended in 1993, and subjects subsequently have been followed annually through the EDIC study (Supplementary Figure 11).

### Genome-wide DNAm measurement, QC, and normalization

Genome-wide DNAm was measured in whole blood by Illumina Infinium Human Methylation EPIC BeadChip array in a subset of DCCT participants (*N* = 499) representing about 125 randomly selected adult subjects from each cohort-treatment group who provided informed consent and had sufficient DNA through a 2-year time period prior to DCCT closeout (Zhuo Chen, Feng Miao, Barbara H Braffett, John M Lachin, Lingxiao Zhang, XiweiWu, Delnaz Roshandel, Melanie Carless, Xuejun Arthur Li, Joshua D Tompkins, JohnKaddis, Arthur D Riggs, Andrew D Paterson, DCCT/EDIC Study Group, RamaNatarajan: DNA methylation: a mediator of HbA1c-associated complicationdevelopment in Type 1 diabetes, Submitted) . DNAm was also measured by Infinium Human Methylation 450K BeadChip array in a smaller subset from the DCCT/EDIC (*N* = 63, 22 overlap with EPIC data) in the whole blood during the same 2-year time period prior to DCCT closeout and in monocytes at EDIC follow-up year 16–17. These included 32 subjects from the conventional treatment group with mean DCCT HbA1c > 9.1% (76 mmol/mol) and significant progression of retinopathy and/or nephropathy from the DCCT closeout to EDIC year 10, and 31 subjects from the intensive treatment group with mean DCCT HbA1c < 7.3% (56 mmol/mol) and no development of retinopathy and nephropathy until EDIC year 10 [41]. Three subjects had missing methylation data for monocytes including two subjects from the conventional and one subject from the intensive treatment group.

The R package meffil (https://github.com/perishky/meffil; accessed on December 2018) [42] was used to perform QC and normalization. Samples were removed if their predicted sex based on DNAm did not match their recorded sex or had > 10% probes with detection *p* value > 0.01 or > 10% probes with bead number < 3. Samples were also excluded if their SNP genotypes did not match with those from GWAS-array (concordance threshold = 0.8). Illumina HumanCoreExome BeadArray (Illumina, San Diego, CA, USA) data imputed to 1000 Genomes (phase 3, v5) was used for this comparison [43]. One sample from EPIC and one sample from 450K monocyte data did not pass QC. Functional normalization (“noob” for dye-bias and background correction followed by “quantile” normalization implemented in meffil) was then performed to account for technical variation in the data [42]. Blood cell proportions were estimated using the method [44] implemented in meffil with “blood gse35069 complete” as reference [42].

### DNAm age calculation

Pan-tissue [1], Skin & Blood [2], PhenoAge [8], and GrimAge [9] were calculated by uploading the data to https://dnamage.genetics.ucla.edu/ (accessed December 2018) with the normalization option selected.

### T1D complications

CVD was described as any CVD from DNA collection date to EDIC follow-up year 20 [45].

Nephropathy was described as first occurrence of sustained (2 consecutive) eGFR < 60 ml/min/1.73 m^2^ [46, 47] or AER ≥ 300 mg/24 h [48] from DCCT closeout to EDIC year 18. The Chronic Kidney Disease Epidemiology Collaboration equation [49] was used to calculate eGFR.

Retinopathy was defined as severe non-proliferative diabetic retinopathy (SNPDR), proliferative diabetic retinopathy (PDR), or clinically significant macular edema (CSME) from DCCT closeout to EDIC follow-up year 18 [50].

Neuropathy was defined as DPN at EDIC year 13/14 and CAN at EDIC year 13/14 and/or EDIC year 16/17 [51].

### Statistical analysis

Spearman correlation was used to test for correlation between chronological age and DNAm age and between the four epigenetic ages. Paired sample *T* tests were used to determine if epigenetic ages were significantly different from chronological age and if the four epigenetic ages differed significantly.

EAA was calculated by subtracting chronological age from epigenetic age (EAA = epigenetic age − chronological age). EPIC array was performed in seven batches. Therefore, batch was included in all multivariable analyses of EPIC data. Since whole blood is a combination of 7 different cell types (neutrophil, B cell, CD4T, CD8T, natural killer cell, eosinophil, and monocyte) and their proportions affect DNAm and epigenetic ages, six predicted cell proportions were included as covariates in all multivariable analyses of whole blood. Neutrophils were excluded as the seven cell proportions sum to one.

Cox proportional hazard models were used to test association of DNAm age with development of CVD, nephropathy, and retinopathy using EPIC data. Logistic regression was used to test association of DNAm age with development of neuropathy during EDIC. Subjects who developed complications during DCCT (before DNAm measurement) were excluded from their corresponding analysis. We also investigated association of DNAm age with repeated measures of renal function, eGFR (annual), and AER (biannual, natural log transformed) from DCCT closeout to EDIC follow-up year 18 using LMMs. Two models were fit for both Cox and LMMs. Model 1 included sex, age, and T1D duration at the time of DNAm measurement along with batch and cell proportions. Model 2 included all covariates in model 1 plus repeated cross-sectional measures of HbA1c (Supplementary Table 12).

In univariable analysis, linear regression was used to test the association of different factors with EAA one at a time. In multivariable analysis, all factors plus chronological age were included in the model, and their associations were tested with DNAm age using linear regression. These factors included sex, cohort, treatment group, and stimulated C-peptide at DCCT eligibility as well as T1D duration, time-weighted HbA1c, BMI, systolic and diastolic blood pressure, HDL, LDL, triglyceride, total cholesterol, pulse rate, current smoking, drinking status, physical activity during work, and leisure time at the time of DNAm measurement (Supplementary Table 3). Since HbA1c and treatment group are highly associated, only HbA1c was included in the multivariable analysis of EPIC data. Due to small sample size and being highly selected on multiple traits, associations of only a subset of factors (sex, age, T1D duration, stimulated C-peptide, HbA1c, cohort, and treatment group) were tested in 450K data.

All the statistical analyses were performed using SAS 9.4 (Cary, NC).

## Supplementary information

**Additional file 1:.**

## Data Availability

DCCT data are available to authorized users at https://repository.niddk.nih.gov and www.ncbi.nlm.nih.gov/projects/gap/cgi-bin/study.cgi?study_id=phs000086.v3.p1. The 450K whole blood and monocyte DNAm datasets are available in the Gene Expression Omnibus (GEO) database, www.ncbi.nlm.nih.gov/geo (accession no. GSE76171). The EPIC DNAm dataset will be uploaded into dbGaP https://www.ncbi.nlm.nih.gov/gap/.

## Abbreviations

AER: Albumin excretion rate
CAN: Cardiovascular autonomic neuropathy
CSME: Clinically significant macular edema
CVD: Cardiovascular diseases
DCCT/EDIC: Diabetes Control and Complications Trial/Epidemiology of Diabetes Interventions and Complications
DNAm: DNA methylation
DPN: Diabetic peripheral neuropathy
EAA: Epigenetic age acceleration
eGFR: estimated glomerular filtration
LMMs: Linear mixed models
PDR: Proliferative diabetic retinopathy
SNPDR: Severe non-proliferative diabetic retinopathy
T1D: Type 1 diabetes
T2D: Type 2 diabetes
WHR: Waist/hip ratio
