# Supplementary material for: DNA methylation age calculators reveal association with diabetic neuropathy in type 1 diabetes
Source: Clin Epigenetics. 2020 Apr 5;12:52. doi: 10.1186/s13148-020-00840-6 (PMC7132894; doi:10.1186/s13148-020-00840-6)
Supplement: Supplementary file 1 — Additional file 1:. [file 13148_2020_840_MOESM1_ESM.docx]

**Supplement**

**Supplementary Tables**

**Supplementary Table 1:** Characteristics of four epigenetic age clocks

| **Clock** | **Illumina Array** | **Tissue/s** | **Train Set** | **Test Set** | **CpGs** | **Target Age** | **Surrogate Markers** | **Method** |
| --- | --- | --- | --- | --- | --- | --- | --- | --- |
| **Pan-tissue (1)** | 27K or 450K | Whole blood  Multiple blood cell types  Cord blood  Brain  Breast (normal)  Buccal  Cartilage Knee  Colon  Dermal fibroblast  Epidermis  Gastric  Head+Neck  Heart  Kidney  Liver  Lung  Bone marrow  Placenta  Prostate (normal)  Saliva  Stomach  Thyroid  Fat  Muscle  placenta  Uterine | 39 datasets  N = 3931 | 32 datasets  N = 3211 | 353 | Chronological | - | Chronological age was regressed on 21,369 CpGs present on 27K and 45K arrays using a penalized regression model (elastic net). |
| **Skin & blood (2)** | 450K and EPIC | Buccal  Whole blood  Epithelium  Fibroblast  Skin  Cord blood  Dermis+Epidermis  Endothelial  Keratinocyte  Lymphoblast  Saliva | 10 datasets  N = 896 | 16 datasets  N = 1326 | 391 | Chronological | - | Chronological age was regressed on CpGs present on 450K and EPIC arrays using a penalized regression model (elastic net). |
| **PhenoAge (3)** | 27k, 450k and EPIC | Whole blood | 1 dataset  N = 9926 | 1 dataset  N = 6209 | 513 | Biological | 1. Albumin 2. Creatinine 3. Glucose, serum 4. C-reactive protein 5. Lymphocyte percent 6. Mean (red) cell volume 7. Red cell distribution width 8. Alkaline phosphatase 9. White blood cell count 10. Age | A Cox penalized regression model was applied where the hazard of aging-related mortality was regressed on 42 clinical markers and chronological age to select variables for inclusion in the phenotypic age score. Ten variables including chronological age were selected for the phenotypic age predictor, and were included in a parametric proportional hazards model to estimate the 10-year mortality risk score. Subsequently, this mortality score was converted into units of years. Finally, the resulting phenotypic age estimate was regressed on DNA methylation data using an elastic net regression analysis. |
| **GrimAge(4)** | 450K and EPIC | Whole blood | 1 dataset  1731 | 1 dataset  625 | 1030  (typically <200 CpGs for pack-years and each of the 7 plasma proteins) | Biological | 1. Adrenomedullin 2. Beta-2-microglobulin 3. Cystatin-C 4. Growth differentiation factor 15 5. Leptin 6. Plasminogen activator inhibitor 1 7. Tissue inhibitor metalloproteinases 1 8. Smoking pack-years | Training data was used to define DNAm-based surrogate markers of 88 plasma protein variables and smoking pack-years. Each of the 88 plasma protein variables was regressed on chronological age, sex, and the CpGs levels in the training data using an elastic net regression model which automatically selected a subset of CpGs whose linear combination best predicted the corresponding plasma Level. Twelve of the 88 plasma proteins and pack-years exhibited a moderately high correlation coefficient between their measured levels and their respective DNAm-based surrogate marker in the test data set.  In the next stage, a predictor of mortality was developed by regressing time-to-death due to all-cause mortality on the DNAm-based estimator of smoking pack-years, chronological age, sex and the 12 DNAm-based surrogate biomarkers of plasma protein levels. The elastic net Cox regression model automatically selected the DNAm pack-years, age, sex, and 7 DNAm-based surrogate markers of plasma proteins.  The linear combination of covariates resulting from the elastic net Cox regression model can be interpreted as an estimate of the logarithm of the hazard ratio of mortality. This parameter was linearly transformed into DNAm GrimAge by performing a linear transformation whose slope and intercept terms were chosen by forcing the mean and variance of GrimAge to match that of chronological age. |

**Supplementary Table 2:** Univariable association of different factors with EAAs in the EPIC dataset

|  | **Pan Tissue EAA** | | **Skin & Blood EAA** | | **PhenoAge EAA** | | **GrimAge EAA** | |
| --- | --- | --- | --- | --- | --- | --- | --- | --- |
| **Factor** | **Beta (SE)** | **P** | **Beta (SE)** | **P** | **Beta (SE)** | **P** | **Beta (SE)** | **P** |
| **Sex (Male)** | 0.86 (0.39) | 2.61E-02 | -0.06 (0.28) | 0.84 | -1.70 (0.48) | 3.76E-04 | 1.90 (0.42) | 6.20E-06 |
| **Cohort (Primary)** | -0.9 (0.38) | 0.02 | -0.60 (0.28) | 3.39E-02 | -0.05 (0.48) | 0.92 | -0.56 (0.42) | 0.19 |
| **Group (Intensive)** | 0.31 (0.39) | 0.41 | 0.35 (0.28) | 0.22 | 0.04 (0.48) | 0.93 | 0.34 (0.42) | 0.42 |
| **Age (years)** | -0.13 (0.03) | 1.18E-04 | 0.07 (0.02) | 6.49E-03 | -0.01 (0.04) | 0.89 | -0.14 (0.04) | 1.03E-04 |
| **T1D Duration (months)** | 0.009 (0.003) | 3.25E-03 | 0.005 (0.002) | 1.72E-02 | 0.008 (0.004) | 3.12E-02 | -0.001 (0.003) | 0.85 |
| **Stimulated C-peptide (pmol/ml)** | -2.75 (1.57) | 0.08 | -1.92 (1.15) | 0.09 | -3.74 (1.95) | 0.06 | -3.13 (1.72) | 0.07 |
| **Time-weighted HbA1c (%)*** | 0.06 (0.15) | 0.69 | -0.11 (0.11) | 0.30 | 0.31 (0.18) | 0.09 | 0.29 (0.16) | 0.07 |
| **BMI (kg/m2)** | 0.01 (0.06) | 0.86 | 0.15 (0.04) | 3.00E-04 | 0.06 (0.07) | 0.42 | -0.06 (0.06) | 0.31 |
| **Systolic blood pressure (mmHg)** | 0.03 (0.02) | 0.14 | 0.01 (0.01) | 0.44 | 0.01 (0.02) | 0.76 | 0.005 (0.018) | 0.79 |
| **Diastolic blood pressure (mmHg)** | 0.03 (0.02) | 0.26 | 0.001 (0.018) | 0.98 | 0.001 (0.03) | 0.98 | -0.03 (0.03) | 0.23 |
| **HDL (mg/dl)** | -0.004 (0.015) | 0.78 | -0.01 (0.01) | 0.32 | 0.03 (0.02) | 0.16 | -0.05 (0.02) | 2.67E-03 |
| **LDL (mg/dl)** | 0.0002 (0.0066) | 0.97 | 0.009 (0.005) | 0.06 | 0.02 (0.01) | 2.80E-02 | 0.008 (0.007) | 0.29 |
| **Triglyceride (mg/dl)** | 0.004 (0.004) | 0.39 | 0.002 (0.003) | 0.47 | -0.002 (0.005) | 0.71 | 0.007 (0.005) | 0.15 |
| **Total cholesterol (mg/dl)** | 0.001 (0.006) | 0.89 | 0.006 (0.004) | 0.15 | 0.02 (0.01) | 1.69E-02 | 0.001 (0.006) | 0.87 |
| **Pulse rate (beat/min)** | -0.001 (0.021) | 0.97 | 0.02 (0.02) | 0.30 | 0.06 (0.03) | 1.75E-02 | 0.02 (0.02) | 0.32 |
| **Current smoker vs. non-smoker** | -0.46 (0.49) | 0.35 | -0.60 (0.36) | 0.09 | 1.09 (0.61) | 0.07 | 7.07 (0.44) | 8.92E-48 |
| **Regular drinker vs. non-drinker** | 0.14 (0.45) | 0.75 | 0.10 (0.33) | 0.76 | 0.08 (0.55) | 0.89 | 1.26 (0.49) | 1.01E-02 |
| **Occasional drinker vs. non-drinker** | -0.25 (0.71) | 0.72 | -0.82 (0.52) | 0.11 | 0.46 (0.88) | 0.60 | 0.09 (0.77) | 0.91 |
| **Strenuous activity vs. sedentary^†^** | -1.23 (1.03) | 0.24 | -1.34 (0.75) | 0.08 | -0.10 (1.28) | 0.94 | 3.86 (1.12) | 5.97E-04 |
| **Moderate activity vs. sedentary^†^** | -0.09 (0.40) | 0.82 | -0.17 (0.29) | 0.56 | 0.44 (0.49) | 0.38 | 0.93 (0.43) | 3.11E-02 |
| **METS >1500 vs. METs <450^‡^** | 0.53 (0.46) | 0.25 | -0.41 (0.34) | 0.22 | 0.34 (0.57) | 0.55 | 0.02 (0.50) | 0.97 |
| **METs 450-1500 vs. METs <450^‡^** | -0.35 (0.48) | 0.47 | -0.83 (0.35) | 1.78E-2 | -1.03 (0.60) | 0.08 | -1.26 (0.52) | 1.65E-2 |

EAA: Epigenetic age acceleration

All factors were obtained at DNAm measurement except for stimulated C-peptide which is measured at DCCT eligibility.

* Time-weighted HbA1c from DCCT baseline to DNAm measurement

**†** Level of activity on the job, at school or in home making: sedentary such as office work with occasional inter-office walking; moderate activity requires considerable but not constant lifting, walking, bending, pulling, etc. such as homemaker with family and without domestic assistance; strenuous activity requires almost constant lifting, bending, pulling, scrubbing, etc. such as furniture mover

**‡** According to the international classification by Ainsworth used by American College of Sports Medicine (ACSM); light, moderate, hard and very hard activity was allocated 3, 4, 6 and 9 METs, respectively. For each participant, these allocated MET value were multiplied by the time (minutes) spent in that activity to obtain the MET for that level of activity. The sum of METs from all activities was recorded as the total leisure time activity for each participant. Subjects then were categorized into three groups based on the ACSM recommendation for METs.min/week (5, 6).

**Supplementary Table 3:** Multivariable association of different factors with EAAs in the EPIC dataset

|  | **Pan Tissue** | | | **Skin & Blood** | | | **PhenoAge** | | | **GrimAge** | | |
| --- | --- | --- | --- | --- | --- | --- | --- | --- | --- | --- | --- | --- |
|  | **β** | **SE** | **P** | **β** | **SE** | **P** | **β** | **SE** | **P** | **β** | **SE** | **P** |
| **Sex (Male)** | 1.17 | 0.37 | 1.85E-03 | -0.40 | 0.27 | 0.15 | -1.72 | 0.45 | 1.71E-04 | 1.72 | 0.41 | 3.53E-05 |
| **Chronological age (years)** | 0.85 | 0.03 | 5.72E-96 | 1.06 | 0.02 | 1.42E-173 | 0.95 | 0.04 | 7.73E-87 | 0.86 | 0.04 | 4.73E-85 |
| **T1D duration (months)** | 0.01 | 0.00 | 4.67E-04 | 0.01 | 0.00 | 1.38E-02 | 0.01 | 0.00 | 2.20E-02 | 0.00 | 0.00 | 0.39 |
| **Time-weighted HbA1c (%)^*^** | 0.04 | 0.14 | 0.75 | -0.02 | 0.10 | 0.82 | 0.45 | 0.16 | 6.67E-03 | 0.32 | 0.15 | 3.16E-02 |

Cell counts (B, CD4T, CD8T, natural killer, eosinophil and monocyte) and batch (as a categorical variable) were also included in the analysis. All factors were obtained at DNAm measurement.

* Time-weighted HbA1c from DCCT baseline to DNAm measurement.

**Supplementary Table 4:** Characteristics of the subjects with 450K data

|  | **Conventional**  **N = 32***  **Mean (SD)/N (%)** | **Intensive**  **N = 31***  **Mean (SD)/N (%)** |
| --- | --- | --- |
| **Sex (Male)** | 15 (46.9%) | 17 (54.8%) |
| **Cohort (Primary)** | 8 (25.0%) | 14 (45.2%) |
| **Age at diagnosis (years)** | 21.0 (7.4) | 23.3 (7.8) |
| **Stimulated C-peptide at DCCT eligibility (pmol/ml)** | 0.09 (0.09) | 0.15 (0.16) |
| **At whole blood measurement** |  |  |
| **Time-weighted HbA1c from DCCT baseline (%)** | 10.19 (0.78) | 6.43 (0.42) |
| **T1D duration (months)** | 142.8 (53.2) | 144.3 (59.0) |
| **Age (years)** | 33.0 (6.6) | 35.3 (6.3) |
| **Pan tissue epigenetic clock** |  |  |
| **DNAm age (years)** | 41.9 (8.1) | 43.2 (7.5) |
| **Epigenetic Age acceleration (years)** | 8.9 (3.5) | 7.9 (4.3) |
| **Skin & blood epigenetic clock** |  |  |
| **DNAm age (years)** | 34.8 (7.7) | 37.0 (7.1) |
| **Epigenetic Age acceleration (years)** | 1.8 (2.9) | 1.7 (3.1) |
| **PhenoAge epigenetic clock** |  |  |
| **DNAm age (years)** | 28.3 (7.8) | 30.35 (10.0) |
| **Epigenetic age acceleration (years)** | -4.7 (4.2) | -4.9 (6.9) |
| **GrimAge epigenetic clock** |  |  |
| **DNAm age (years)** | 39.3 (6.3) | 40.8 (6.2) |
| **Epigenetic age acceleration (years)** | 6.3 (2.4) | 5.5 (3.1) |
| **At monocyte measurement** |  |  |
| **Time-weighted HbA1c from DCCT baseline (%)** | 8.85 (0.88) | 6.71 (0.65) |
| **T1D duration (months)** | 351.4 (55.9) | 356.0 (58.2) |
| **Age (years)** | 49.8 (6.8) | 52.5 (6.4) |
| **Pan tissue epigenetic clock** |  |  |
| **DNAm age (years)** | 56.1 (6.7) | 56.0 (6.6) |
| **Epigenetic age acceleration (years)** | 6.3 (4.2) | 3.5 (4.6) |
| **Skin & blood epigenetic clock** |  |  |
| **DNAm age (years)** | 52.6 (6.3) | 54.7 (6.2) |
| **Epigenetic age acceleration (years)** | 2.8 (3.6) | 2.2 (3.6) |
| **PhenoAge epigenetic clock** |  |  |
| **DNAm age (years)** | 58.0 (7.8) | 60.7 (9.2) |
| **Epigenetic age acceleration (years)** | 8.2 (5.6) | 8.2 (9.8) |
| **GrimAge epigenetic clock** |  |  |
| **DNAm age (years)** | 54.6 (6.6) | 56.8 (6.4) |
| **Epigenetic age acceleration (years)** | 4.8 (3.3) | 4.3 (3.3) |

* Three subjects had missing data at monocyte measurement; 2 from conventional and 1 from intensive treatment group.

**Supplementary Table 5:** Correlation and mean difference between epigenetic ages and chronological age in whole blood 450K data

|  | **Chronological Age** | **Pan Tissue** | **Skin & Blood** | **PhenoAge** | **GrimAge** |
| --- | --- | --- | --- | --- | --- |
| **Chronological Age** |  | 0.86 | 0.91 | 0.79 | 0.92 |
| **Pan Tissue** | 8.4 (4.0) |  | 0.89 | 0.84 | 0.81 |
| **Skin & Blood** | 1.7 (3.0) | -6.7 (3.6) |  | 0.83 | 0.87 |
| **PhenoAge** | -4.8 (0.7) | -13.2 (4.7) | -6.6 (5.1) |  | 0.78 |
| **GrimAge** | 5.9 (0.3) | -2.5 (4.6) | 4.2 (3.8) | 10.7 (6.7) |  |

The values above the diagonal are Spearman correlation coefficients. The values below the diagonal are mean differences (mean of the age in the row – mean of the age in the column) and their corresponding SDs in brackets. All p-values regarding the correlations and the mean differences are < 0.0001.

**Supplementary Table 6:** Univariable association of different factors with EAAs in whole blood 450K dataset

|  | **Pan Tissue EAA** | | **Skin & Blood EAA** | | **PhenoAge EAA** | | **GrimAge EAA** | |
| --- | --- | --- | --- | --- | --- | --- | --- | --- |
| **Factor** | **Beta (SE)** | **P** | **Beta (SE)** | **P** |  |  | **Beta (SE)** | **P** |
| **Sex (Male)** | -0.15 (1.01) | 0.88 | -1.1 (0.75) | 0.15 | -1.94 (1.41) | 0.17 | 0.84 (0.7) | 0.23 |
| **Cohort (Primary)** | -0.97 (1.05) | 0.36 | -0.78 (0.79) | 0.33 | -1.31 (1.50) | 0.38 | 0.53 (0.73) | 0.47 |
| **Group (Intensive)** | -1.07 (1.00) | 0.29 | -0.05 (0.76) | 0.95 | -0.23 (1.43) | 0.87 | -0.78 (0.7) | 0.27 |
| **Age (years)** | 0.02 (0.08) | 0.75 | 0.04 (0.06) | 0.45 | 0.06 (0.11) | 0.56 | -0.13 (0.05) | 1.46E-2 |
| **T1D Duration (months)** | 0.01 (0.01) | 0.16 | 0.01 (0.01) | 0.29 | 0.01 (0.01) | 0.25 | -0.002 (0.006) | 0.72 |
| **Stimulated C-peptide (pmol/ml) *** | -4.62 (3.85) | 0.23 | -0.81 (2.95) | 0.78 | -5.49 (5.51) | 0.32 | 1.16 (2.72) | 0.67 |
| **Time-weighted HbA1c (%) †** | 0.31 (0.25) | 0.23 | -0.02 (0.19) | 0.92 | 0.02 (0.37) | 0.96 | 0.25 (0.18) | 0.16 |

EAA: Epigenetic age acceleration

* Stimulated C-peptide at DCCT eligibility

**†** Time-weighted HbA1c from DCCT baseline to monocyte DNAm measurement.

**Supplementary Table 7:** Correlation and mean difference between epigenetic ages and chronological age in monocyte 450K data

|  | **Chronological Age** | **Pan Tissue** | **Skin & Blood** | **PhenoAge** | **GrimAge** |
| --- | --- | --- | --- | --- | --- |
| **Chronological Age** |  | 0.77 | 0.85 | 0.72 | 0.89 |
| **Pan Tissue** | 4.9 (4.6) |  | 0.82 | 0.75 | 0.74 |
| **Skin & Blood** | 2.5 (3.6) | -2.4 (3.6) |  | 0.80 | 0.79 |
| **PhenoAge** | 8.2 (6.2) | 3.3 (5.5) | 5.7 (5.2) |  | 0.70 |
| **GrimAge** | 4.5 (2.3) | -0.4 (5.0)^*^ | 2.0 (4.2) | -3.7 (6.7) |  |

The values above the diagonal are Spearman correlation coefficients. The values below the diagonal are mean differences (mean of the age in the row – mean of the age in the column) and their corresponding SDs in brackets. All p-values regarding the correlations and the mean differences are < 0.001 except the one with * which the p-value is not significant.

**Supplementary Table 8:** Univariable association of different factors with EAAs in monocyte 450K dataset

|  | **Pan Tissue EAA** | | **Skin & Blood EAA** | | **PhenoAge EAA** | | **GrimAge EAA** | |
| --- | --- | --- | --- | --- | --- | --- | --- | --- |
| **Factor** | **Beta (SE)** | **P** | **Beta (SE)** | **P** | **Beta (SE)** | **P** | **Beta (SE)** | **P** |
| **Sex (Male)** | 2.44 (1.15) | 3.87E-2 | -0.54 (0.94) | 0.57 | -2.42 (1.58) | 0.13 | 1.23 (0.85) | 0.15 |
| **Cohort (Primary)** | -1.77 (1.23) | 0.15 | -0.31 (0.98) | 0.75 | -1.07 (1.68) | 0.53 | 1.46 (0.88) | 0.1 |
| **Group (Intensive)** | -2.86 (1.13) | 1.44E-2 | -0.59 (0.93) | 0.53 | 0 (1.6) | 1.00 | -0.5 (0.86) | 0.56 |
| **Age (years)** | -0.25 (0.08) | 5.21E-3 | -0.2 (0.07) | 3.73E-3 | -0.1 (0.12) | 0.39 | -0.18 (0.06) | 3.81E-3 |
| **T1D Duration (months)** | 0 (0.01) | 0.69 | 0 (0.01) | 0.76 | 0 (0.01) | 0.82 | -0.01 (0.01) | 0.06 |
| **Stimulated C-peptide (pmol/ml) *** | -14.07 (4.27) | 1.69E-3 | -4.59 (3.6) | 0.21 | -9.54 (6.12) | 0.12 | 2.85 (3.33) | 0.39 |
| **Time-weighted HbA1c (%) †** | 0.67 (0.48) | 0.16 | 0.1 (0.38) | 0.80 | -0.1 (0.65) | 0.88 | 0.29 (0.35) | 0.4 |

EAA: Epigenetic age acceleration

* Stimulated C-peptide at DCCT eligibility

**†** Time-weighted HbA1c from DCCT baseline to monocyte DNAm measurement.

**Supplementary Table 9:** Characteristics of the DCCT/EDIC subjects with and without EPIC data

|  | **With EPIC Data** | **Without EPIC Data** |  |
| --- | --- | --- | --- |
|  | **N = 499** | **N = 920** |  |
|  | **Mean (SD)/N (%)** | **Mean (SD)/N (%)** | **p** |
| **Sex (Male)** | 271 (54.3%) | 474 (51.5%) | 0.32 |
| **Stimulated C-peptide at DCCT Eligibility (pmol/ml)** | 0.12 (0.12) | 0.11 (0.11) | 0.24 |
| **Time-weighted HbA1c (%)*** | 7.93 (1.35) | 8.29 (1.53) | 9.54E-5 |
| **Current smoker** | 95 (19.0%) | 181 (19.7%) | 0.77 |
| **Alcohol consumption** |  |  |  |
| **None** | 326 (65.3%) | 598 (65%) |  |
| **Occasional** | 42 (8.4%) | 89 (9.7%) | 0.9 |
| **Regular** | 131 (26.3%) | 233(25.3%) |  |
| **Physical activity job^†^** |  |  |  |
| **Sedentary** | 210 (42.1%) | 401 (43.6%) |  |
| **Moderate** | 270 (54.1%) | 484 (52.6%) | 0.63 |
| **Strenuous** | 19 (3.8%) | 35 (3.8%) |  |
| **Leisure time physical activity^‡^** |  |  |  |
| **METs <450** | 175 (37.1%) | 289 (31.9%) | 0.21 |
| **METs 450-1500** | 148 (29.7%) | 288 (31.3%) |  |
| **METs >1500** | 175 (35.1%) | 342 (37.2%) |  |
| **Missing** | 1 (0.2%) | 1 (0.1%) |  |
| **BMI (kg/m2)** | 25.45 (3.35) | 25.61 (3.53) | 0.54 |
| **Systolic blood pressure (mmHg)** | 114.35 (11.2) | 115.47 (11.85) | 0.21 |
| **Diastolic blood pressure (mmHg)** | 73.59 (8.07) | 74.49 (8.81) | 3.64E-2 |
| **HDL (mg/dl)** | 51.89 (12.85) | 51.55 (12.87) | 0.7 |
| **LDL (mg/dl)** | 111.23 (29.09) | 113.27 (29.77) | 0.23 |
| **Triglyceride (mg/dl)** | 78.57 (45.51) | 87.44 (54.81) | 1.45E-3 |
| **Total cholesterol (mg/dl)** | 178.76 (32.91) | 182.34 (34.69) | 4.61E-2 |
| **Pulse rate (beat/min)** | 72.14 (9.39) | 74.92 (10.72) | 5.21E-6 |
| **Age at diagnosis (years)** | 23.15 (7.21) | 20.16 (8.38) | 2.43E-10 |
| **Duration (months)** | 133.1 (61.16) | 134.46 (56.96) | 0.33 |
| **Age (years)** | 34.2 (5.72) | 31.36 (7.43) | 8.09E-12 |

All factors were obtained at DNAm measurement except for stimulated C-peptide which is measured at DCCT eligibility.

Chi-Square test was used for comparison of sex and current smoker in the two groups. Cochran-Armitage trend test was used for comparison of alcohol consumption, and physical activity at job and leisure time physical activity in the two groups. Wilcoxon-Mann-Whitney test was used for comparison of all continues variables (C-peptide, HbA1c, BMI, systolic and diastolic blood pressure, lipid levels, pulse pressure, age at diagnosis, duration and age) in the 2 groups.

* Time-weighted HbA1c since DCCT baseline.

**†** Level of activity on the job, at school or in home making: sedentary such as office work with occasional inter-office walking; moderate activity requires considerable but not constant lifting, walking, bending, pulling, etc. such as homemaker with family and without domestic assistance; strenuous activity requires almost constant lifting, bending, pulling, scrubbing, etc. such as furniture mover

**‡** According to the international classification by Ainsworth used by American College of Sports Medicine (ACSM); light, moderate, hard and very hard activity was allocated 3, 4, 6 and 9 METs, respectively. For each participant, these allocated MET value were multiplied by the time (minutes) spent in that activity to obtain the MET for that level of activity. The sum of METs from all activities was recorded as the total leisure time activity for each participant. Subjects then were categorized into three groups based on the ACSM recommendation for METs.min/week (5, 6).

**Supplementary Table 10:** Characteristics of the DCCT/EDIC subjects in primary cohort/conventional treatment group with and without EPIC data

|  | **With EPIC Data** | **Without EPIC Data** |  |
| --- | --- | --- | --- |
|  | **N = 124** | **N = 250** |  |
|  | **Mean (SD)/N (%)** | **Mean (SD)/N (%)** | **p** |
| **Sex (Male)** | 73 (58.9%) | 131 (52.4%) | 0.24 |
| **Stimulated C-peptide at DCCT Eligibility (pmol/ml)** | 0.18 (0.14) | 0.16 (0.12) | 0.18 |
| **Time-weighted HbA1c (%)*** | 8.94 (1.23) | 9.32 (1.38) | 1.61E-2 |
| **Current smoker** | 24 (19.4%) | 45 (18.0%) | 0.75 |
| **Alcohol consumption** |  |  |  |
| **None** | 81 (65.3%) | 164 (65.6%) |  |
| **Occasional** | 11 (8.9%) | 20 (8.0%) | 0.97 |
| **Regular** | 32 (25.8%) | 66 (26.4%) |  |
| **Physical activity at job^†^** |  |  |  |
| **Sedentary** | 57 (46.0%) | 108 (43.2%) |  |
| **Moderate** | 64 (51.6%) | 132 (52.8%) | 0.49 |
| **Strenuous** | 3 (2.4%) | 10 (4.0%) |  |
| **Leisure time physical activity^‡^** |  |  |  |
| **METs <450** | 38 (30.7%) | 72 (28.8%) | 0.63 |
| **METs 450-1500** | 39 (31.5%) | 78 (31.2%) |  |
| **METs >1500** | 46 (37.1%) | 99 (39.6%) |  |
| **Missing** | 1 (0.8%) | 1 (0.4%) |  |
| **BMI (kg/m2)** | 24.85 (2.77) | 24.72 (3.04) | 0.60 |
| **Systolic blood pressure (mmHg)** | 112.64 (10.73) | 113.2 (11.65) | 0.72 |
| **Diastolic blood pressure (mmHg)** | 73.9 (8.21) | 73.85 (8.18) | 0.91 |
| **HDL (mg/dl)** | 52.9 (13.02) | 52.06 (13.14) | 0.58 |
| **LDL (mg/dl)** | 107.72 (30.21) | 112.94 (30.91) | 0.13 |
| **Triglyceride (mg/dl)** | 75.27 (41.14) | 90.18 (53.5) | 2.667E-3 |
| **Total cholesterol (mg/dl)** | 175.66 (34.35) | 183.03 (36.09) | 4.08E-2 |
| **Pulse rate (beat/min)** | 70.94 (9.76) | 73.96 (10.72) | 2.14E-2 |
| **Age at diagnosis (years)** | 26.52 (5.91) | 22.21 (8.35) | 1.06E-5 |
| **Duration (months)** | 88.03 (28.14) | 92.77 (29.01) | 0.15 |
| **Age (years)** | 33.84 (6.12) | 29.94 (7.72) | 9.91E-6 |

All factors were obtained at DNAm measurement except for stimulated C-peptide which is measured at DCCT eligibility.

Chi-Square test was used for comparison of sex and current smoker in the two groups. Cochran-Armitage trend test was used for comparison of alcohol consumption and leisure time physical activity in the two groups. Cochran-Armitage trend exact test was used for comparison of physical activity at job in the two groups. Wilcoxon-Mann-Whitney test was used for comparison of all continues variables (C-peptide, HbA1c, BMI, systolic and diastolic blood pressure, lipid levels, pulse pressure, age at diagnosis, duration and age) in the 2 groups.

* Time-weighted HbA1c since DCCT baseline.

**†** Level of activity on the job, at school or in home making: sedentary such as office work with occasional inter-office walking; moderate activity requires considerable but not constant lifting, walking, bending, pulling, etc. such as homemaker with family and without domestic assistance; strenuous activity requires almost constant lifting, bending, pulling, scrubbing, etc. such as furniture mover

**‡** According to the international classification by Ainsworth used by American College of Sports Medicine (ACSM); light, moderate, hard and very hard activity was allocated 3, 4, 6 and 9 METs, respectively. For each participant, these allocated MET value were multiplied by the time (minutes) spent in that activity to obtain the MET for that level of activity. The sum of METs from all activities was recorded as the total leisure time activity for each participant. Subjects then were categorized into three groups based on the ACSM recommendation for METs.min/week (5, 6).

**Supplementary Table 11:** Characteristics of the DCCT/EDIC subjects in primary cohort/intensive treatment group with and without EPIC data

|  | **With EPIC Data** | **Without EPIC Data** |  |
| --- | --- | --- | --- |
|  | **N = 125** | **N = 216** |  |
|  | **Mean (SD)/N (%)** | **Mean (SD)/N (%)** | **p** |
| **Sex (Male)** | 60 (48.0%) | 108 (50.0%) | 0.74 |
| **Stimulated C-peptide at DCCT Eligibility (pmol/ml)** | 0.17 (0.14) | 0.15 (0.13) | 0.41 |
| **Time-weighted HbA1c (%)*** | 6.93 (0.72) | 7.29 (1.02) | 1.91E-3 |
| **Current smoker** | 19 (15.2%) | 45 (20.8%) | 0.25 |
| **Alcohol consumption** |  |  |  |
| **None** | 80 (64.0%) | 135 (62.5%) |  |
| **Occasional** | 12 (9.6%) | 27 (12.5%) | 0.99 |
| **Regular** | 33 (26.4%) | 54 (25.0%) |  |
| **Physical activity at job^†^** |  |  |  |
| **Sedentary** | 49 (39.2%) | 92 (42.6%) |  |
| **Moderate** | 69 (55.5%) | 118 (54.6%) | 0.36 |
| **Strenuous** | 7 (5.6%) | 6 (2.8%) |  |
| **Leisure time physical activity^‡^** |  |  |  |
| **METs <450** | 47 (37.6%) | 54 (25.0%) | 1.96E-2 |
| **METs 450-1500** | 34 (27.2%) | 66 (30.6%) |  |
| **METs >1500** | 44 (35.2%) | 96 (44.4%) |  |
| **Missing** | 0 (0%) | 0 (0%) |  |
| **BMI (kg/m2)** | 25.42 (3.29) | 26.46 (3.78) | 3.14E-2 |
| **Systolic blood pressure (mmHg)** | 112.27 (11.37) | 115.56 (10.6) | 7.68E-3 |
| **Diastolic blood pressure (mmHg)** | 72.29 (7.69) | 73.81 (8.67) | 0.06 |
| **HDL (mg/dl)** | 54.86 (14.71) | 51.51 (12.46) | 0.06 |
| **LDL (mg/dl)** | 111.49 (26.95) | 108.25 (29.2) | 0.27 |
| **Triglyceride (mg/dl)** | 74.15 (45.07) | 81.99 (57.47) | 0.24 |
| **Total cholesterol (mg/dl)** | 181.15 (31.31) | 176.21 (32.44) | 0.25 |
| **Pulse rate (beat/min)** | 71.23 (9.05) | 73.31 (11.09) | 0.12 |
| **Age at diagnosis (years)** | 26.85 (5.43) | 22.54 (8.13) | 7.95E-6 |
| **Duration (months)** | 85.7 (23.92) | 95.46 (29.44) | 1.82E-3 |
| **Age (years)** | 33.95 (5.59) | 30.52 (7.65) | 1.16E-4 |

All factors were obtained at DNAm measurement except for stimulated C-peptide which is measured at DCCT eligibility.

Chi-Square test was used for comparison of sex and current smoker in the two groups. Cochran-Armitage trend test was used for comparison of alcohol consumption and leisure time physical activity in the two groups. Cochran-Armitage trend exact test was used for comparison of physical activity at job in the two groups. Wilcoxon-Mann-Whitney test was used for comparison of all continues variables (C-peptide, HbA1c, BMI, systolic and diastolic blood pressure, lipid levels, pulse pressure, age at diagnosis, duration and age) in the 2 groups.

* Time-weighted HbA1c since DCCT baseline.

**†** Level of activity on the job, at school or in home making: sedentary such as office work with occasional inter-office walking; moderate activity requires considerable but not constant lifting, walking, bending, pulling, etc. such as homemaker with family and without domestic assistance; strenuous activity requires almost constant lifting, bending, pulling, scrubbing, etc. such as furniture mover

**‡** According to the international classification by Ainsworth used by American College of Sports Medicine (ACSM); light, moderate, hard and very hard activity was allocated 3, 4, 6 and 9 METs, respectively. For each participant, these allocated MET value were multiplied by the time (minutes) spent in that activity to obtain the MET for that level of activity. The sum of METs from all activities was recorded as the total leisure time activity for each participant. Subjects then were categorized into three groups based on the ACSM recommendation for METs.min/week (5, 6).

**Supplementary Table 12:** Characteristics of the DCCT/EDIC subjects in secondary cohort/conventional treatment group with and without EPIC data

|  | **With EPIC Data** | **Without EPIC Data** |  |
| --- | --- | --- | --- |
|  | **N = 125** | **N = 223** |  |
|  | **Mean (SD)/N (%)** | **Mean (SD)/N (%)** | **p** |
| **Sex (Male)** | 65 (52.0%) | 120 (53.8%) | 0.75 |
| **Stimulated C-peptide at DCCT Eligibility (pmol/ml)** | 0.08 (0.09) | 0.07 (0.08) | 3.60E-2 |
| **Time-weighted HbA1c (%)*** | 8.72 (1.19) | 9.1 (1.31) | 6.28E-3 |
| **Current smoker** | 25(20.0%) | 47 (21.1%) | 0.81 |
| **Alcohol consumption** |  |  |  |
| **None** | 83 (66.4%) | 139 (62.3%) |  |
| **Occasional** | 8 (6.4%) | 22 (9'9%) | 0.64 |
| **Regular** | 34 (27.2%) | 62 (27.8%) |  |
| **Physical activity at job^†^** |  |  |  |
| **Sedentary** | 49 (39.2%) | 92 (41.3%) |  |
| **Moderate** | 74 (59.2%) | 120 (53.8%) | 0.84 |
| **Strenuous** | 2 (1.6%) | 11 (4.9%) |  |
| **Leisure time physical activity^‡^** |  |  |  |
| **METs <450** | 38 (30.4%) | 74 (33.2%) |  |
| **METs 450-1500** | 41 (32.8%) | 71 (31.8%) | 0.62 |
| **METs >1500** | 46 (36.8%) | 78 (35.0%) |  |
| **BMI (kg/m2)** | 24.97 (2.83) | 25.11 (3.12) | 0.71 |
| **Systolic blood pressure (mmHg)** | 116.2 (11.53) | 117.26 (13.03) | 0.76 |
| **Diastolic blood pressure (mmHg)** | 73.78 (8.14) | 75.81 (9.18) | 2.38E-2 |
| **HDL (mg/dl)** | 51.03 (11.35) | 50.46 (12) | 0.57 |
| **LDL (mg/dl)** | 114.54 (30.96) | 117.43 (31.02) | 0.43 |
| **Triglyceride (mg/dl)** | 88.14 (54.21) | 88.85 (50.09) | 0.80 |
| **Total cholesterol (mg/dl)** | 182.96 (34.6) | 185.64 (36.59) | 0.61 |
| **Pulse rate (beat/min)** | 72.86 (9.29) | 76.91 (10.51) | 3.32E-4 |
| **Age at diagnosis (years)** | 19.78 (7.08) | 17.53 (7.84) | 1.64E-2 |
| **Duration (months)** | 173.46 (51.8) | 176.18 (48.05) | 0.79 |
| **Age (years)** | 34.12 (5.3) | 32.22 (6.88) | 1.28E-2 |

All factors were obtained at DNAm measurement except for stimulated C-peptide which is measured at DCCT eligibility.

Chi-Square test was used for comparison of sex and current smoker in the two groups. Cochran-Armitage trend test was used for comparison of alcohol consumption and leisure time physical activity in the two groups. Cochran-Armitage trend exact test was used for comparison of physical activity at job in the two groups. Wilcoxon-Mann-Whitney test was used for comparison of all continues variables (C-peptide, HbA1c, BMI, systolic and diastolic blood pressure, lipid levels, pulse pressure, age at diagnosis, duration and age) in the 2 groups.

* Time-weighted HbA1c since DCCT baseline.

**†** Level of activity on the job, at school or in home making: sedentary such as office work with occasional inter-office walking; moderate activity requires considerable but not constant lifting, walking, bending, pulling, etc. such as homemaker with family and without domestic assistance; strenuous activity requires almost constant lifting, bending, pulling, scrubbing, etc. such as furniture mover

**‡** According to the international classification by Ainsworth used by American College of Sports Medicine (ACSM); light, moderate, hard and very hard activity was allocated 3, 4, 6 and 9 METs, respectively. For each participant, these allocated MET value were multiplied by the time (minutes) spent in that activity to obtain the MET for that level of activity. The sum of METs from all activities was recorded as the total leisure time activity for each participant. Subjects then were categorized into three groups based on the ACSM recommendation for METs.min/week (5, 6).

**Supplementary Table 13:** Characteristics of the DCCT/EDIC subjects in secondary cohort/intensive treatment group with and without EPIC data

|  | **With EPIC Data** | **Without EPIC Data** |  |
| --- | --- | --- | --- |
|  | **N = 125** | **N = 231** |  |
|  | **Mean (SD)/N (%)** | **Mean (SD)/N (%)** | **p** |
| **Sex (Male)** | 73 (58.4%) | 115 (49.8%) | 0.12 |
| **Stimulated C-peptide at DCCT Eligibility (pmol/ml)** | 0.06 (0.06) | 0.06 (0.08) | 0.50 |
| **Time-weighted HbA1c (%)*** | 7.14 (0.75) | 7.31 (0.96) | 0.31 |
| **Current smoker** | 27 (21.6%) | 44 (19.1%) | 0.56 |
| **Alcohol consumption** |  |  |  |
| **None** | 82 (65.6%) | 160 (69.3%) |  |
| **Occasional** | 11 (8.8%) | 20 (8.7%) | 0.44 |
| **Regular** | 32 (25.6%) | 51 (22.1%) |  |
| **Physical activity at job^†^** |  |  |  |
| **Sedentary** | 55 (44.0%) | 106 (47.2%) |  |
| **Moderate** | 63 (50.4%) | 114 (49.4%) | 0.44 |
| **Strenuous** | 7 (5.6%) | 8 (3.5%) |  |
| **Leisure time physical activity^‡^** |  |  |  |
| **METs <450** | 52 (41.6%) | 89 (38.5%) |  |
| **METs 450-1500** | 34 (27.2%) | 73 (31.6%) | 0.85 |
| **METs >1500** | 39 (31.2%) | 69 (29.9%) |  |
| **BMI (kg/m2)** | 26.56 (4.09) | 26.25 (3.84) | 0.53 |
| **Systolic blood pressure (mmHg)** | 116.27 (10.64) | 116.09 (11.68) | 0.41 |
| **Diastolic blood pressure (mmHg)** | 74.41 (8.15) | 74.54 (9.12) | 0.76 |
| **HDL (mg/dl)** | 48.77 (11.37) | 52.09 (13.73) | 4.07E-2 |
| **LDL (mg/dl)** | 111.14 (28.02) | 114.3 (27.19) | 0.22 |
| **Triglyceride (mg/dl)** | 76.69 (39.26) | 88.22 (57.96) | 0.07 |
| **Total cholesterol (mg/dl)** | 175.25 (30.92) | 184.14 (32.74) | 1.03E-2 |
| **Pulse rate (beat/min)** | 73.5 (9.31) | 75.54 (10.27) | 0.06 |
| **Age at diagnosis (years)** | 19.47 (6.66) | 18.26 (7.98) | 0.16 |
| **Duration (months)** | 184.84 (48.41) | 175.76 (47.91) | 0.10 |
| **Age (years)** | 34.89 (5.87) | 32.87 (7.05) | 1.40E-2 |

All factors were obtained at DNAm measurement except for stimulated C-peptide which is measured at DCCT eligibility.

Chi-Square test was used for comparison of sex and current smoker in the two groups. Cochran-Armitage trend test was used for comparison of alcohol consumption and leisure time physical activity in the two groups. Cochran-Armitage trend exact test was used for comparison of physical activity at job in the two groups. Wilcoxon-Mann-Whitney test was used for comparison of all continues variables (C-peptide, HbA1c, BMI, systolic and diastolic blood pressure, lipid levels, pulse pressure, age at diagnosis, duration and age) in the 2 groups.

* Time-weighted HbA1c since DCCT baseline.

**†** Level of activity on the job, at school or in home making: sedentary such as office work with occasional inter-office walking; moderate activity requires considerable but not constant lifting, walking, bending, pulling, etc. such as homemaker with family and without domestic assistance; strenuous activity requires almost constant lifting, bending, pulling, scrubbing, etc. such as furniture mover

**‡** According to the international classification by Ainsworth used by American College of Sports Medicine (ACSM); light, moderate, hard and very hard activity was allocated 3, 4, 6 and 9 METs, respectively. For each participant, these allocated MET value were multiplied by the time (minutes) spent in that activity to obtain the MET for that level of activity. The sum of METs from all activities was recorded as the total leisure time activity for each participant. Subjects then were categorized into three groups based on the ACSM recommendation for METs.min/week (5, 6).

**Supplementary Figures**

**Supplementary Figure 1:** The CpGs shared among the 3 epigenetic clocks


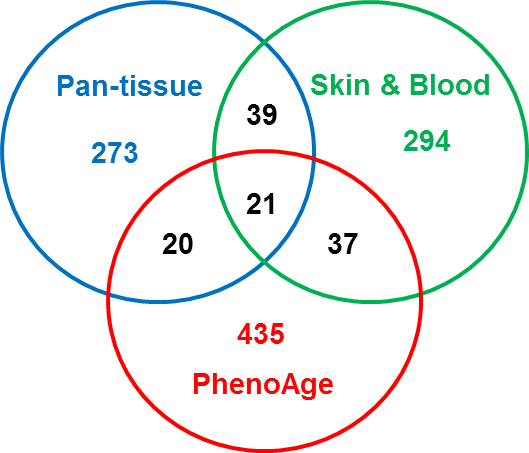


GrimAge CpGs are not publicly available. Therefore, they were not included in the plot.

**Supplementary Figure 2:** Epigenetic ages vs. each other in EPIC data

**
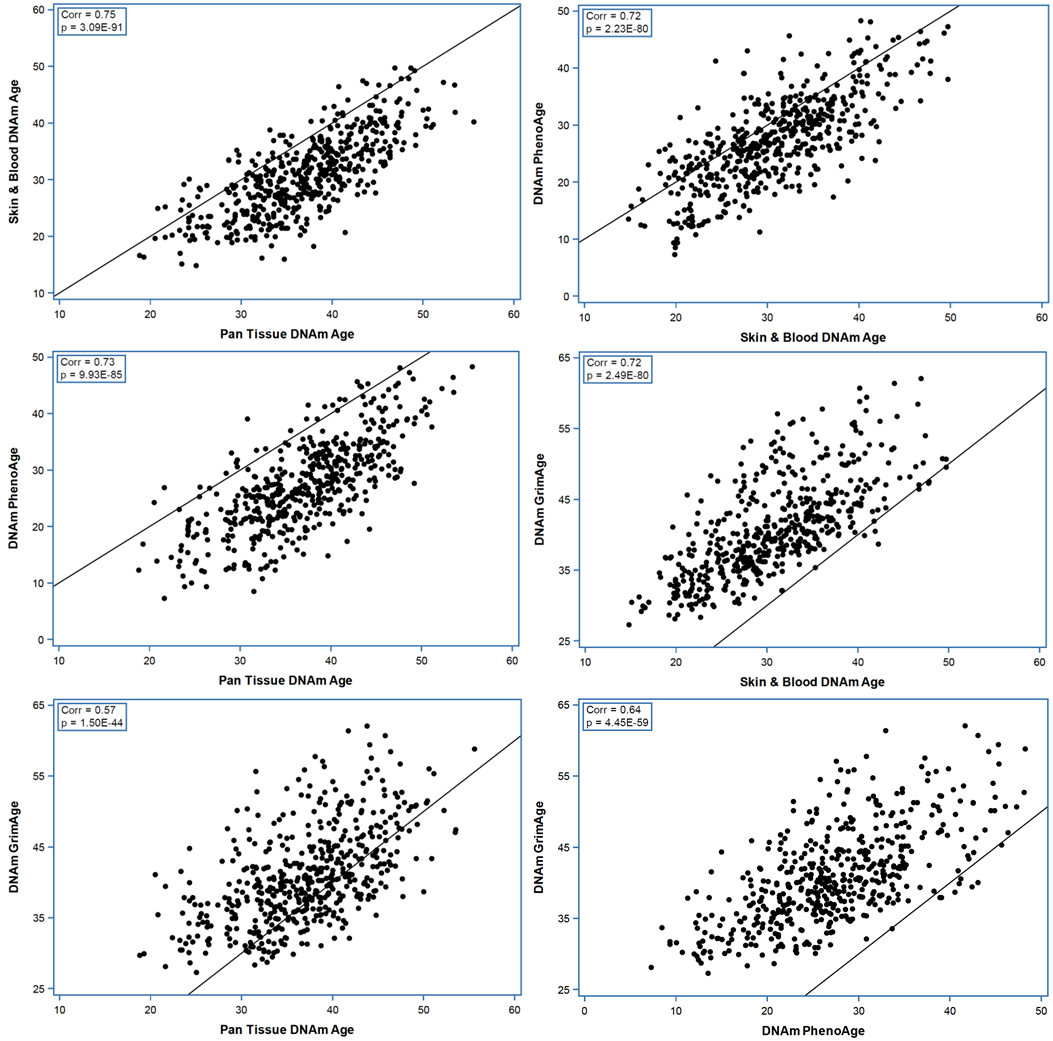
**

Corr: Spearman correlation coefficient. The line is X=Y.

**Supplementary Figure 3:** Association of PhenoAge with AER (natural log transformed) in different EDIC follow-up years


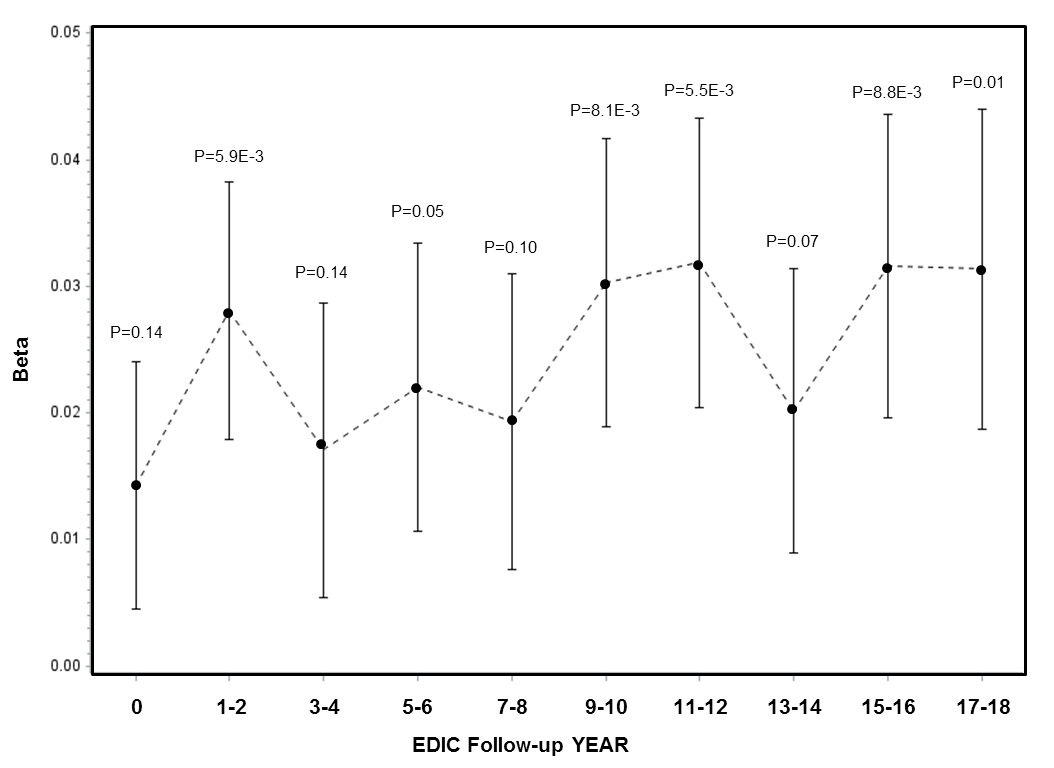


AER was measured for half of the subjects in each follow-up year (i.e. each subjects has AER every 2 years). Therefore, the association of PhenoAge with AER (natural log transformed) was tested and shown for every 2 follow-up year using linear regression. Sex, age and T1D duration at DNA measurement, plus batch and cell proportions were included in the model as covariates. Everybody has AER at baseline.

Beta refers to increase in natural log transformed AER per one-year increase in PhenoAge. The bars are showing ±SE.

**Supplementary Figure 4:** Association of GrimAge with AER (natural log transformed) in different EDIC follow-up years


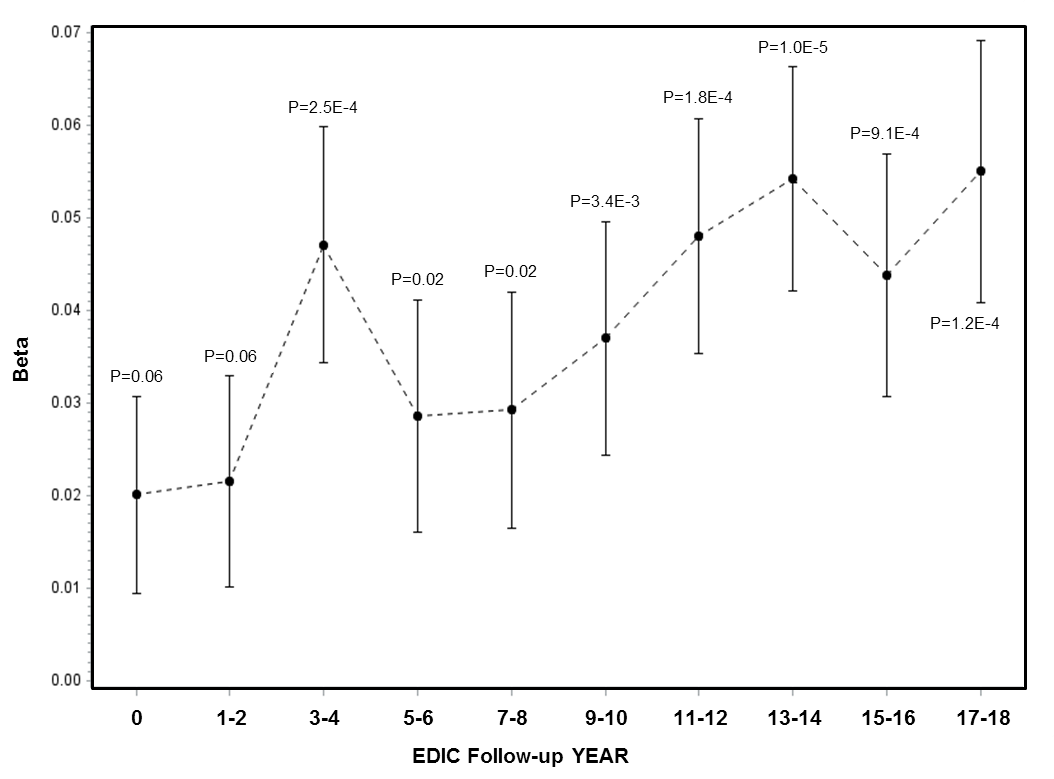


AER was measured for half of the subjects in each follow-up year (i.e. each subjects has AER every 2 years). Therefore, the association of GrimAge with AER (natural log transformed) was tested and shown for every 2 follow-up years using linear regression. Sex, age and T1D duration at DNA measurement, plus batch and cell proportions were included in the model as covariates. Everybody has AER at baseline.

Beta refers to increase in natural log transformed AER per one-year increase in PhenoAge. The bars are showing ±SE.

Supplementary Figure 5: Epigenetic ages vs. chronological age in whole blood 450K data


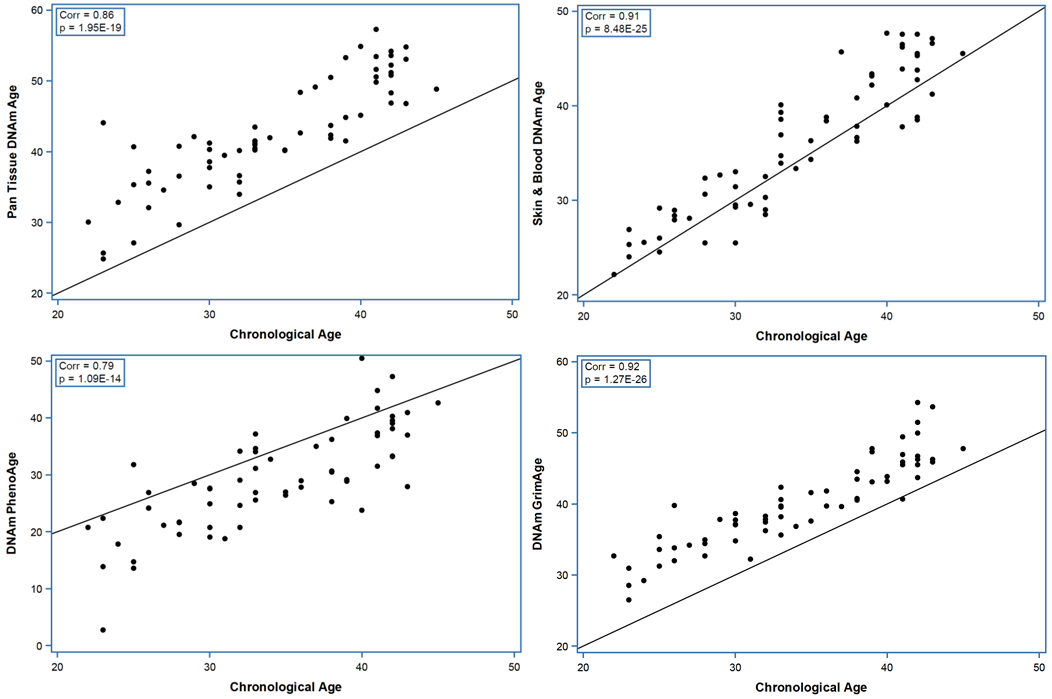


Corr: Spearman correlation coefficient. The line is X =Y.

**Supplementary Figure 6:** Epigenetic ages vs. each other in whole blood 450K data

**
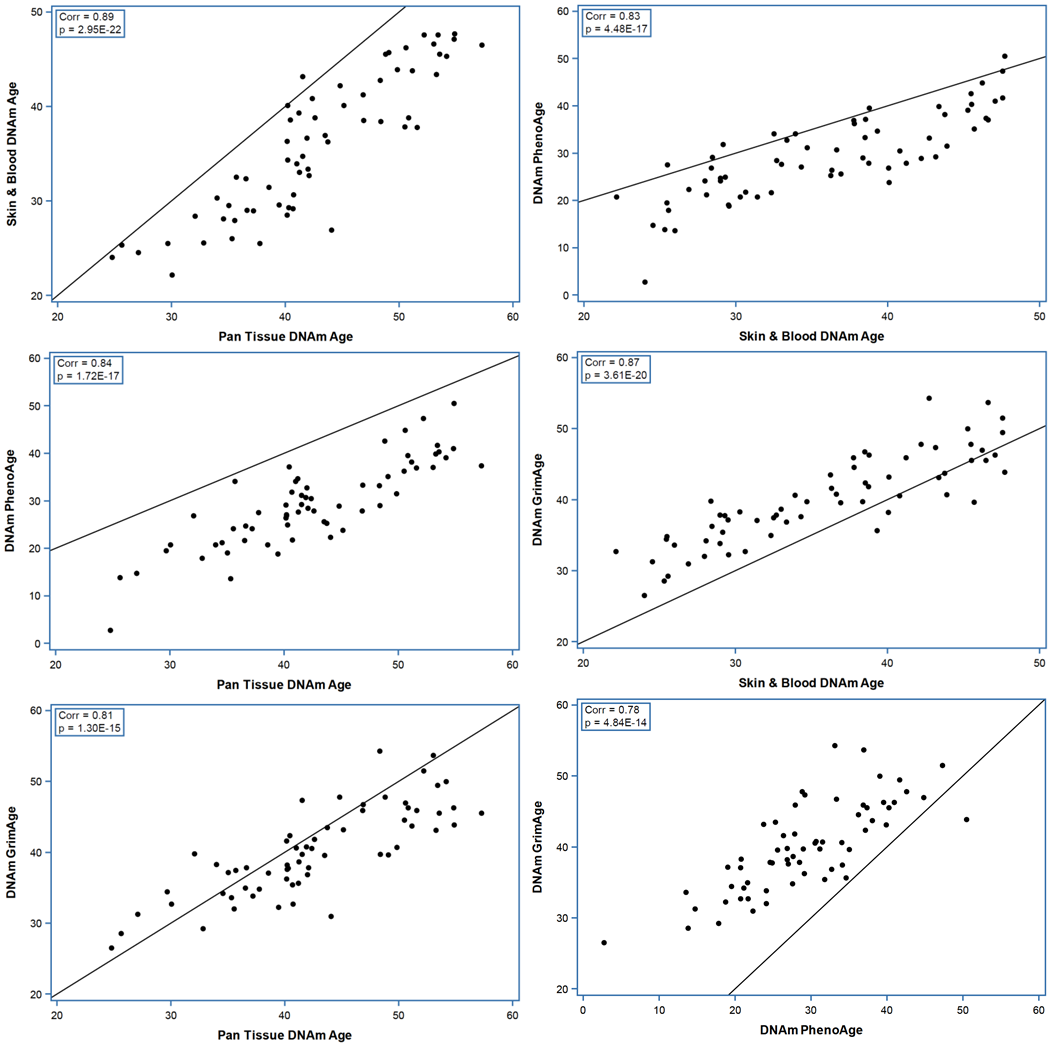
**

Corr: Spearman correlation coefficient. The line is X = Y.

**Supplementary Figure 7:** Epigenetic age acceleration vs. chronological age in whole blood 450K data


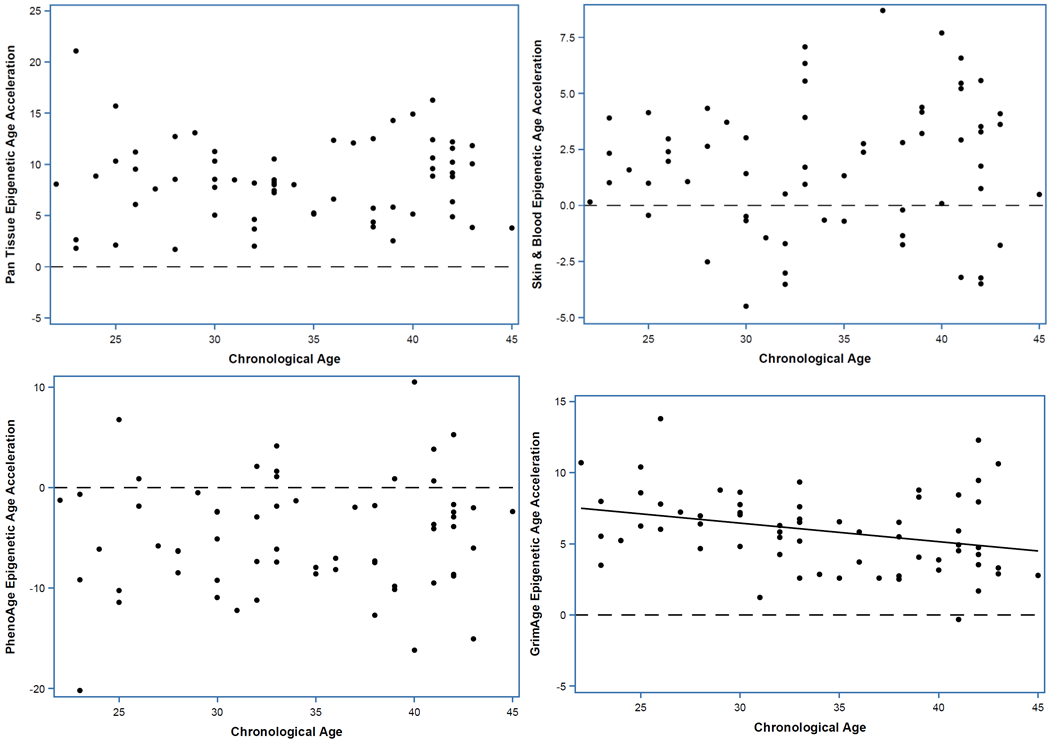


The dash line is age acceleration = 0. The solid line is the line fitting linear regression model with chronological age as predictor and epigenetic age acceleration as outcome, and is present when chronological age is significantly associated with epigenetic age acceleration (p <0.05).

Supplementary Figure 8: Epigenetic ages vs. chronological age in monocytes 450K data


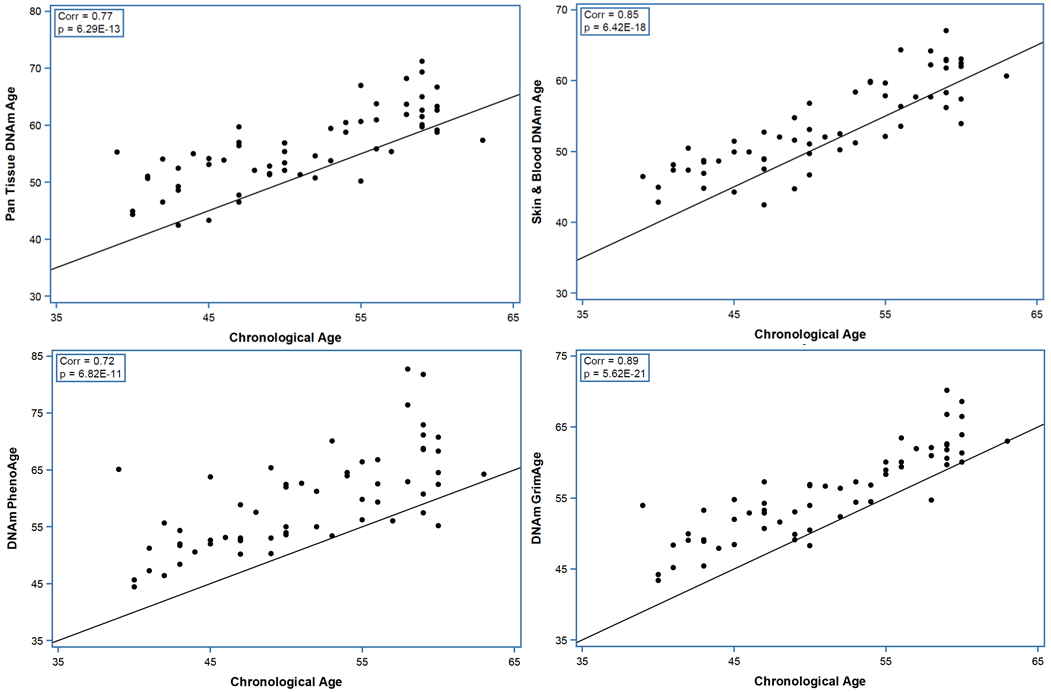


Corr: Spearman correlation coefficient. The line is X=Y.

**Supplementary Figure 9:** Epigenetic ages vs. each other in monocytes 450K data


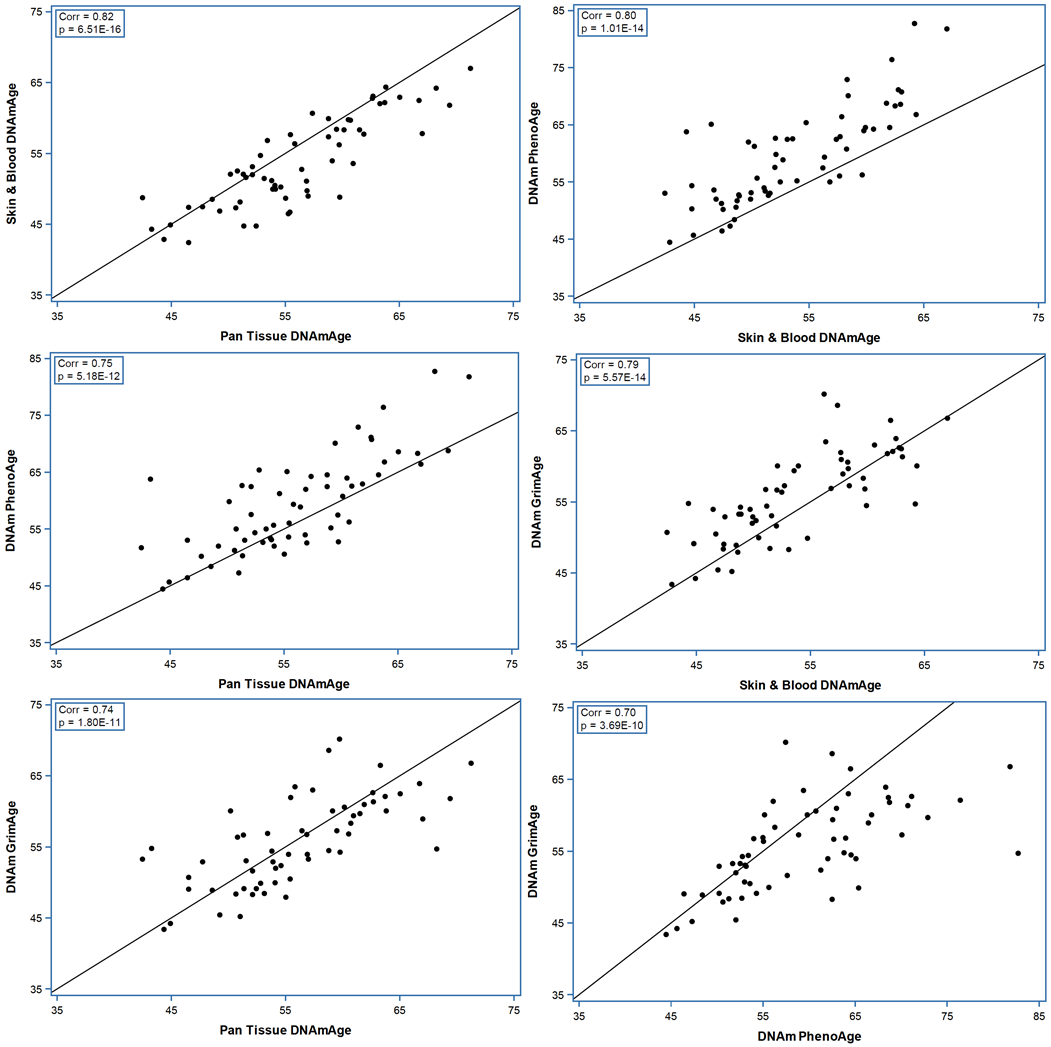


Corr: Spearman correlation coefficient. The line is X axis = Y axis.

**Supplementary Figure 10:** Epigenetic age acceleration vs. chronological age in monocytes 450K data


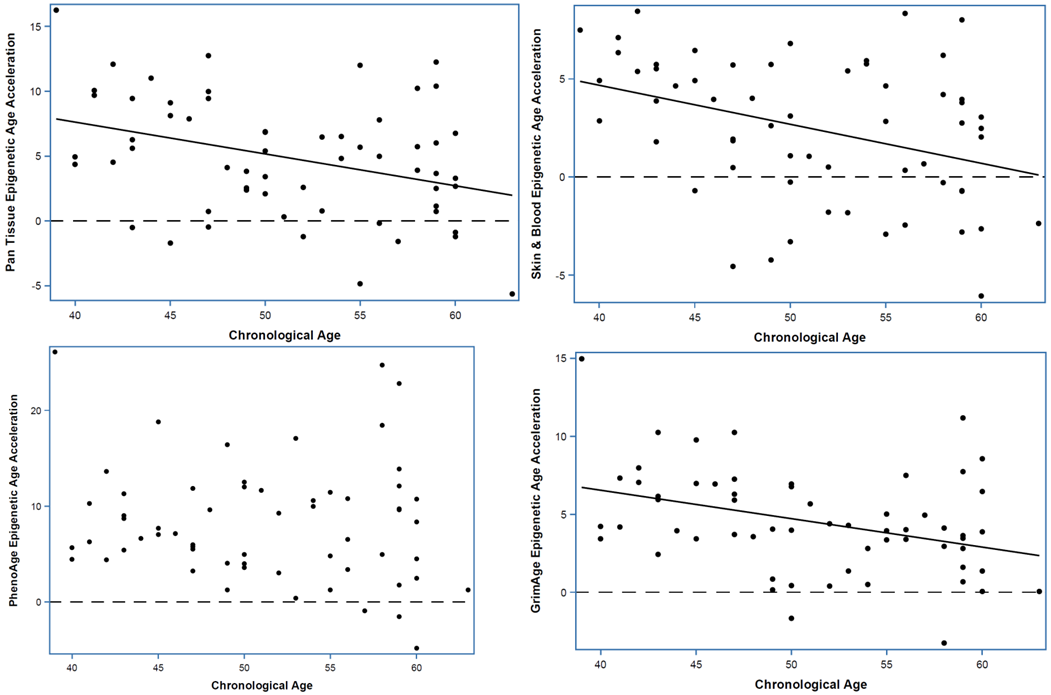


The dash line is epigenetic age acceleration = 0. The solid line is the line fitting linear regression model with chronological age as predictor and epigenetic age acceleration as outcome, and is present when chronological age is significantly associated with epigenetic age acceleration (p <0.05).

**Supplementary Figure 11:** Study design
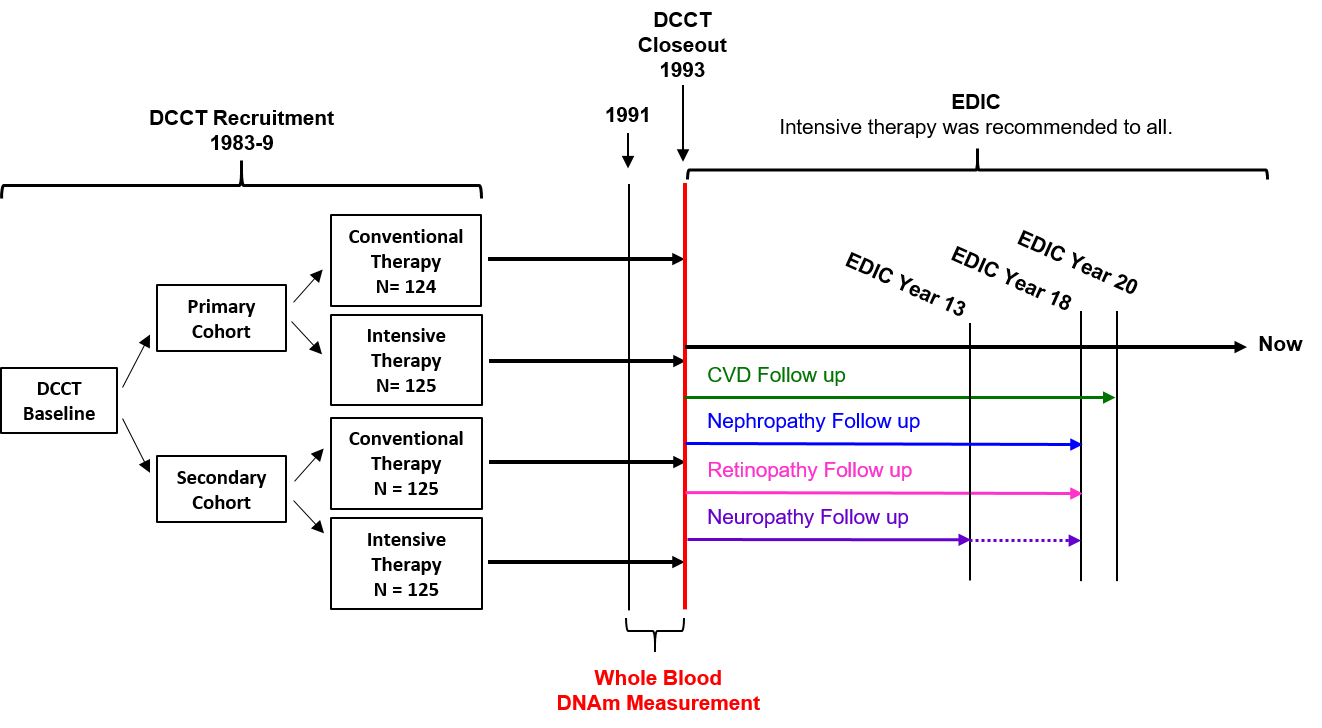


DNAm: DNA methylation, CVD: Cardiovascular diseases

**Supplementary Figure 12:** Analysis plan


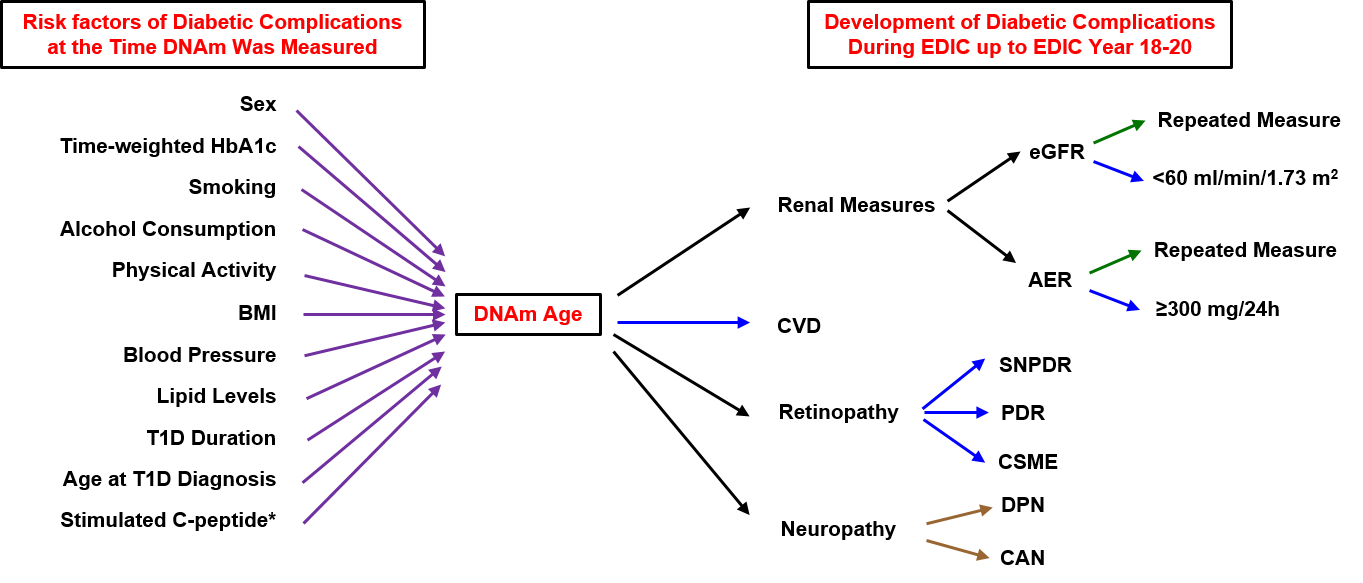


DNAm: DNA methylation, CVD: Cardiovascular diseases, SNPDR: Severe non-proliferative diabetic retinopathy, PDR: Proliferative diabetic retinopathy, CSME: Clinically significant macular edema, AER: Albumin excretion rate, eGFR: estimated glomerular filtration, DPN: Diabetic peripheral neuropathy, CAN: Cardiovascular autonomic neuropathy

* Stimulated C-peptide was measured at DCCT baseline.

The purple, green, blue and brown arrows refer to linear regression models, linear mixed models, Cox proportional hazard models and logistic regression, respectively.

**References:**

1. Horvath S. DNA methylation age of human tissues and cell types. Genome Biol. 2013;14(10):R115.

2. Horvath S, Oshima J, Martin GM, Lu AT, Quach A, Cohen H, et al. Epigenetic clock for skin and blood cells applied to Hutchinson Gilford Progeria Syndrome and ex vivo studies. Aging (Albany NY). 2018;10(7):1758-75.

3. Levine ME, Lu AT, Quach A, Chen BH, Assimes TL, Bandinelli S, et al. An epigenetic biomarker of aging for lifespan and healthspan. Aging (Albany NY). 2018;10(4):573-91.

4. Lu AT, Quach A, Wilson JG, Reiner AP, Aviv A, Raj K, et al. DNA methylation GrimAge strongly predicts lifespan and healthspan. Aging (Albany NY). 2019;11(2):303-27.

5. Makura CB, Nirantharakumar K, Girling AJ, Saravanan P, Narendran P. Effects of physical activity on the development and progression of microvascular complications in type 1 diabetes: retrospective analysis of the DCCT study. BMC Endocr Disord. 2013;13:37.

6. Haskell WL, Lee IM, Pate RR, Powell KE, Blair SN, Franklin BA, et al. Physical activity and public health: updated recommendation for adults from the American College of Sports Medicine and the American Heart Association. Med Sci Sports Exerc. 2007;39(8):1423-34.
